# Antibiotics-induced monodominance of a novel gut bacterial order

**DOI:** 10.1136/gutjnl-2018-317715

**Published:** 2019-01-18

**Authors:** Falk Hildebrand, Lucas Moitinho-Silva, Sonja Blasche, Martin T Jahn, Toni Ingolf Gossmann, Jaime Huerta-Cepas, Rajna Hercog, Mechthild Luetge, Mohammad Bahram, Anna Pryszlak, Renato J Alves, Sebastian M Waszak, Ana Zhu, Lumeng Ye, Paul Igor Costea, Steven Aalvink, Clara Belzer, Sofia K Forslund, Shinichi Sunagawa, Ute Hentschel, Christoph Merten, Kiran Raosaheb Patil, Vladimir Benes, Peer Bork

**Affiliations:** 1 Structural and Computational Biology Unit, European Molecular Biology Laboratory, Heidelberg, Germany; 2 RD3 Marine Microbiology, GEOMAR Helmholtz Centre for Ocean Research Kiel, Kiel, Germany; 3 Department of Animal and Plant Sciences, University of Sheffield, Sheffield, UK; 4 Computational systems biology and genomics, Centro de Biotecnología y Genómica de Plantas, Universidad Politécnica de Madrid (UPM)-Instituto Nacional de Investigación y Tecnología Agraria y Alimentaria (INIA), Madrid, Spain; 5 Genomics Core Facility, European Molecular Biology Laboratory, Heidelberg, Germany; 6 Department of Ecology, Swedish University of Agricultural Sciences, Uppsala, Sweden; 7 Department of Botany, Institute of Ecology and Earth Sciences, Tartu University, Tartu, Estonia; 8 Structural and Computational Biology Unit, Joint PhD degree between EMBL and Heidelberg University, Heidelberg, Germany; 9 Genome Biology Unit, European Molecular Biology Laboratory, Heidelberg, Germany; 10 Host Microbiota Interactions Laboratory, Wellcome Trust Sanger Institute, Wellcome Genome Campus, Hinxton, UK; 11 Biotechnology Dept., GenScript Corporation (NanJing), NanJing, China; 12 Institute of Microbiology, Wagenigen University, Wageningen, Netherlands; 13 Experimental and ClinicalResearch Centre, Max Delbrück Centre for Molecular Medicine, Berlin, Germany; 14 Department of Biology, Institute of Microbiology, ETH Zurich, Zurich, Switzerland; 15 Molecular Medicine Partnership Unit (MMPU), University of Heidelberg and European Molecular Biology Laboratory, Heidelberg, Germany; 16 Department of Bioinformatics, University of Würzburg, Würzburg, Germany

**Keywords:** antibiotics, bacterial overgrowth, intestinal microbiology, molecular genetics

## Abstract

**Objective:**

The composition of the healthy human adult gut microbiome is relatively stable over prolonged periods, and representatives of the most highly abundant and prevalent species have been cultured and described. However, microbial abundances can change on perturbations, such as antibiotics intake, enabling the identification and characterisation of otherwise low abundant species.

**Design:**

Analysing gut microbial time-series data, we used shotgun metagenomics to create strain level taxonomic and functional profiles. Community dynamics were modelled postintervention with a focus on conditionally rare taxa and previously unknown bacteria.

**Results:**

In response to a commonly prescribed cephalosporin (ceftriaxone), we observe a strong compositional shift in one subject, in which a previously unknown species, ^U^ *Borkfalki ceftriaxensis*, was identified, blooming to 92% relative abundance. The genome assembly reveals that this species (1) belongs to a so far undescribed order of Firmicutes, (2) is ubiquitously present at low abundances in at least one third of adults, (3) is opportunistically growing, being ecologically similar to typical probiotic species and (4) is stably associated to healthy hosts as determined by single nucleotide variation analysis. It was the first coloniser after the antibiotic intervention that led to a long-lasting microbial community shift and likely permanent loss of nine commensals.

**Conclusion:**

The bloom of ^U^ *B. ceftriaxensis* and a subsequent one of *Parabacteroides distasonis* demonstrate the existence of monodominance community states in the gut. Our study points to an undiscovered wealth of low abundant but common taxa in the human gut and calls for more highly resolved longitudinal studies, in particular on ecosystem perturbations.

**Significance of the study**

**What is already known on the subject?**

- Antibiotic treatments are known to reduce bacterial diversity short term, recovering usually within weeks.
- Antibiotic-associated diarrhoea can be associated to blooms of pathogenic bacteria, like *Clostridium difficile*, although the cause is often also unknown.
- Most abundant bacteria of the human gut microbiome are characterised and sequenced, but much less is known about the rare biosphere.

**What are the new findings?**

- A member of an undiscovered order of Firmicutes that is prevalent in one-third of human gut samples, bloomed in multiple individuals after treatment with antibiotics. During blooming, it rose to 92% relative abundance, corresponding to a 2000-fold increase in absolute abundance.
- The extreme bloom uncovers for the first-time monodominant states in the adult gut by commensal bacteria. This was further reinforced by a second commensal, *Parabacteroides distasonis*, also becoming monodominant at 95% relative abundance.
- Nine commensal bacteria are absent in all time points after the extreme community reshaping, further confirmed using complete genome mappings.
- A recurrent bacterial network response is observed after cephalosporin antibiotic treatment.

**How might it impact on clinical practice in the foreseeable future?**

- This study highlights the detrimental impact of antibiotics on the adult gut microbiome, as well as shedding light on the recolonisation process, leading to ecosystem insights into antibiotic side effects.

## Introduction

The gut microbiota plays an important role in human health^1 2^ and is characterised by a diverse community and complex genetic variation landscape deducible from shotgun metagenomics data.^3^ Compared with the skin or oral cavity, GI microbiomes are remarkably stable over prolonged periods,^4^ which in particular applies to the more highly abundant species.^5^ Much less attention has, however, been paid to low abundant species, despite having important keystone functions in the human gut^6^ and explaining most of the temporal variation through conditionally rare taxa (CRT).^7^ The abundance range is usually limited and extreme relative abundances exceeding 60% are only defined for forest ecosystems as monodominance.^8^ Nonetheless, monodominance can be observed, for example, during the first days after birth in human gut microbiomes^9^ or in adults during infectious diarrhoea (typically associated to Proteobacteria such as *Vibrio cholera* or *Escherichia coli* 10). Frequently, however, the infectious source of diarrhoea remains unidentified,^11^ and little is known of the role of CRTs in the recolonisation of the gut microbiome after exogenous disturbances, such as antibiotic treatments.

This knowledge gap is compounded by CRTs often not being represented in genome collections^12^ or ill-defined. CRTs are often not detectable in metagenomes, because they are per definition rare and therefore difficult to spot with standard metagenomic approaches that rely on having either a reference genome in databases^13 14^ or sufficient read coverage in multiple samples to de novo construct metagenomic assembled genomes (MAGs).^15^ But even with reconstructed MAGs, insights on the ecological role of these novel species remain usually limited as the functional annotation of MAGs is often automatic.

To investigate microbial dynamics in response to antibiotic perturbations and the influence of CRTs, we leveraged an extensive longitudinal sampling and respective metagenome sequencing^16^ (see online supplementary file 1). We found that a novel bacterial species, ^U^ *Borkfalki ceftriaxensis*, which belongs to a new order of Firmicutes and bloomed during the first 3 days after antibiotic treatment to monodominant levels. ^U^ *B. ceftriaxensis* is thus the first report of a gut conditionally monodominant taxa (CMT) that is not invasive. Instead, it is globally prevalent in the population, but only associated to humans in our analysis of >3500 metagenomic samples. The initial colonisation by this species was succeeded by a second monodominant state by *Parabacteroides distasonis*. Of two antibiotics treatments, the first ceftriaxone treatment led to long-lasting community composition changes, including the apparent loss of nine species. Our study illustrates the wide range of abundance dynamics in the human gut microbiome and reveals that low abundant species can have important roles in community structuring.

10.1136/gutjnl-2018-317715.supp1 Supplementary file 1

## Results

### ^U^ *B. ceftriaxensis* is a novel, common and low abundant human gut species

We used a metagenomic time series of one individual, HD.S1, sampled biannually over the course of 4 years, with a denser sampling around three interventions (two antibiotic treatments starting on day 370 and day 875 and a bowel cleansing event on day 630). Part of this time series was reported before^16^ and was extended by 10 samples to a total of 24 time points, with some samples being resequenced to greater depth or read length (see onlinesupplementary file 1). All samples were subjected to deep shotgun metagenomics (mean 68 million paired-end reads per sample). During the first intravenous antibiotics treatment on day 370, subject HD.S1 was hospitalised, receiving the third-generation cephalosporin, ceftriaxone. The sample from the day after the end of the antibiotic treatment (HD.S1.374, day 374) was excluded in previous analysis as it contained mostly reads that were not assignable to known species using standard approaches (e.g., mOTU or metaphlan^13 14^). Here, we performed de novo assembly of HD.S1.374 and metagenomic gene-level analysis, which both showed a single, hitherto unknown species, dominating this sample at 92% relative abundance (online supplementary figure 1). Attempts to culture this species were not successful (see online supplementary tables 1 and 2), but we were able to assemble the species’ genome with a quality comparable to assemblies from cultured species (see online supplementary tables 3 and 4). The conserved 16S rRNA gene sequence was very divergent (≤86% nucleotide (nt) identity) to known bacteria (see the ^U^B. *ceftriaxensis* belongs to a new order of Firmicutes section). Since the genome assembly is based solely on metagenomics, we named it ^U^ *B. ceftriaxensis* gen. nov., sp. nov., following the nomenclature proposed for uncultured species.^17^

10.1136/gutjnl-2018-317715.supp2 Supplementary file 2

**Figure 1:**
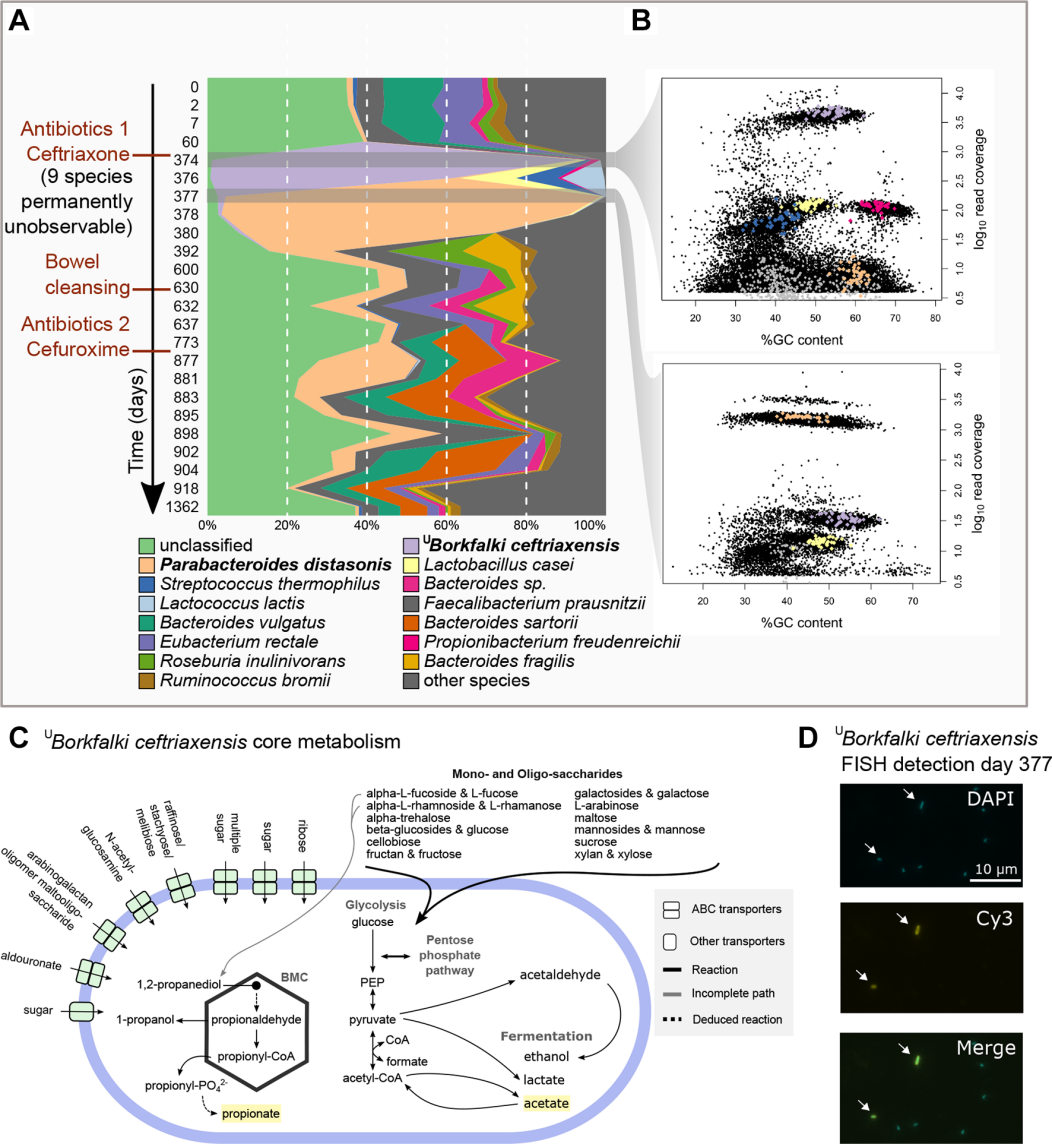
Discovery and tracking of a novel bacterial species. (A) Composition plot (proportional abundance) of the 14 most abundant species in subject HD.S1 over the experiment duration. (B) The faecal microbial community of subject HD.S1 on days 374 and 377 after antibiotic treatment was characterised by extreme low diversity. The graphs show all genes (coverage >3) assembled from these sample with their guanine-cytosine (GC) content and coverage (online supplementary figures 16 and 17). Each sample is dominated by a single species and visible as denser gene clouds (38-fold and 47-fold higher abundance then second most abundant species in those samples). The coloured dots represent the conserved marker genes^29^ of five species (colours as in A). The grey squares show 40 conserved marker genes of other species that are very low abundant. (C) Summary of functional potential of ^U^ *Borkfalki ceftriaxensis* based on genome assembly and functional annotation (see online supplementary file 1). Short-chain fatty acids are indicated by yellow background. (D) ^U^ *B. ceftriaxensis* in faecal samples was detected via FISH, showing that it is viable within stool samples. It is a rod-shaped bacterium (white arrows, see online supplementary figure 15 for additional details). The tested sample was HD.S1.377. BMC, bacterial microcompartment, FISH, fluorescence in situ hybridisation.

Using fluorescence in situ hybridisation (FISH) (see online supplementary file 1), we could detect ^U^ *B. ceftriaxensis* in faecal samples (figure 1D), demonstrating that it was present in the stool. Labelled cells were rod shaped and on average 1.5 (±0.3)×0.8 (± 0.1) μm long and wide (online supplementary table 5). Functional analysis of its gene content indicated that it is a facultative anaerobe spore-forming fermenter, with a large spectrum of monosaccharide and oligosaccharide utilisation genes (figure 1C, online supplementary figure 2 and table 6 for a complete description). Its predicted fermentation products include two of the three short-chain fatty acids, acetate and propionate.

10.1136/gutjnl-2018-317715.supp4 Supplementary file 4

The discovery of ^U^ *B. ceftriaxensis* was enabled by its bloom after beta-lactam ceftriaxone treatment. We identified two genes distantly related to beta-lactamases, but in vitro tests showed only modest activity (see online supplementary file 1). Instead, it seems most likely that ^U^ *B. ceftriaxensis* is inherently resilient to ceftriaxone, due to its penicillin binding proteins of classes known to confer resistance in gram positive bacteria (see online supplement discussion) and due to its spore-forming ability.^18^ Its predicted diversified carbohydrate metabolism makes it plausible that ^U^ *B. ceftriaxensis* can grow extremely fast in an ecosystem devoid of competition.

To further explore this species’ global prevalence, we used the assembled genome as a bait to enable sensitive homology searches against 3692 public metagenomes (online supplementary table 7). We detected this species in most other samples from subject HD.S1, but also in 35% of adult human faecal samples (n=1558), as well as in 5% of samples from other body sites (n=972). Despite this wide prevalence, ^U^ *B. ceftriaxensis* was present at very low abundance (median 0.08%, max 0.7% relative abundance excluding samples from subject HD.S1, see online supplementary file 1 for additional details). It was further not detected in 1163 metagenomes balanced among ocean, soil and animal (mouse, pig, cat and dog) guts, strongly suggesting that ^U^ *B. ceftriaxensis* is a commensal specific to humans and mostly thrives in the gut microbiome, although at usually low abundances.

10.1136/gutjnl-2018-317715.supp7 Supplementary file 7

### ^U^ *B. ceftriaxensis* is stably associated with its hosts

To investigate if ^U^ *B. ceftriaxensis* is a resident commensal of the healthy gut microbiome, we investigated if it is present over time in the same individual. Indeed, the species can be detected in multiple time points of the same individual (10 of 45 analysed individuals, online supplementary tables 7 and 8). In the 24 time-series samples of subject HD.S1, it was detected in eight of these, spanning 1 year before and after the ceftriaxone treatment (online supplementary figure 1). Species level dynamics might be blurred by the influx of conspecific strains with different gene content,^19^ as exchanges of dominant strains within a species have been reported in relation to external interventions.^20 21^ Yet, we hypothesised that the same strain of ^U^ *B. ceftriaxensis* was already a stable resident before the intervention, as subject HD.S1 did not report adverse effects after the antibiotic treatment and it seems more likely that the immune system would tolerate known commensals.^22^

**Figure 2:**
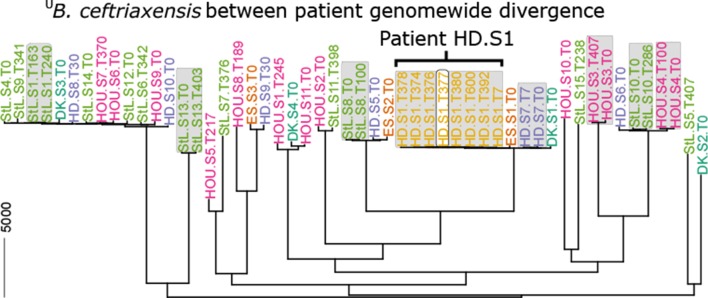
^U^ *Borkfalki ceftriaxensis* is found in multiple samples, but there is a clear substructure to the population. (A) Genotypes of ^U^ *B. ceftriaxensis* in 37 patients based on all genomic sites, using samples with ≥2× genome coverage. Genotypes are in all cases specific to a subject where time series samples are available, with grey boxes indicating the same host. ^U^ *B. ceftriaxensis* type strain HDS1380 is marked by a white box. ^U^ *B. ceftriaxensis* shows large variation between individuals. The scale bar (5000 nt) corresponds to 0.2% genomic divergence.

To investigate strain dynamics, we reconstructed the ^U^ *B. ceftriaxensis* genotype for 57 samples (37 individuals, including eight with ≥2 time points) that had >2× genome coverage as lower limit (mean 4.2× vertical and 71% horizontal genome coverage; online supplementary figure 3), using a novel single nucleotide variant (SNV) caller adapted for low abundant species (see online supplementary file 1). ^U^ *B. ceftriaxensis* had a clear population substructure, although being closely related: the mean between-person genotype evolutionary similarity was 99.2%±0.34%, based on a recombination-corrected phylogeny^23^; therefore, all strains seem to belong to the same ecotype.^24^ This phylogeny showed that all strains without exception remain over time most similar within their specific host (figure 2, grey boxes), implying ^U^ *B. ceftriaxensis* is a stable part of the human microbiome. Furthermore, we found no evidence of multiple ^U^ *B. ceftriaxensis* strains colonising the same host (online supplementary figure S4C). However, some individuals carry very closely related strains. For example, genotypes of subjects HD.S7, ES.S1 and DK.S1 are on average only 185 nt distant from that of subject HD.S1 across the whole genome, suggesting the existence of a very recent progenitor of these strains. Nonetheless, even within this group, the genomes from single individuals can be clearly separated (figure 2).

**Figure 3:**
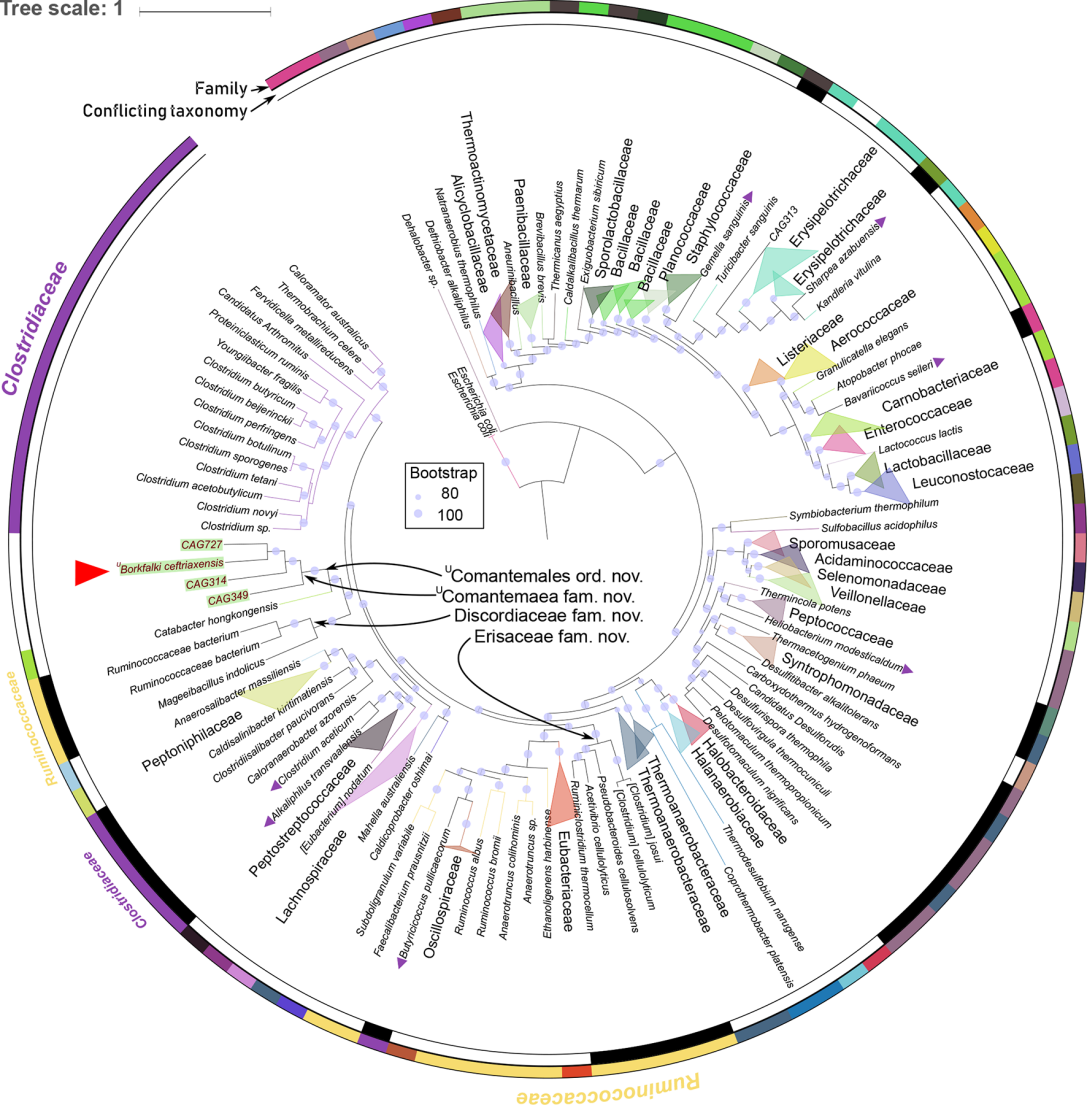
Phylogenetic maximum likelihood tree of all Firmicutes genera in reference database, based on the AA sequence of 40 conserved genes. ^U^ *Borkfalki ceftriaxensis* placement (red triangle) suggests that it represents a novel family, ^U^Comantemaea, as well as a new order, ^U^Comantemales, in phylum Firmicutes. Three uncharacterised putative species (MAGs) also belong to this family that were obtained by metagenomic binning before.^15^ Only non-parametric bootstrap values >80/100 are shown (blue circles). Novel proposed Ruminococcaceae families are shown with arrows, to split this family into monophyletic groups. The outer ring colour shows family assignments of the remaining taxa in the tree. All monophyletic families are collapsed, while remaining families were either paraphyletic or the placement seemed not to fit a monophyletic family origin. Further, species that group in conflict to current naming schemes are marked with purple triangles (online supplementary table 10). The tree is rooted with *Escherichia coli*, a gamma-proteobacterium. CAG, co-abundance group; IS, incertae sedis; MAG, metagenomic assembled genomes.

**Figure 4:**
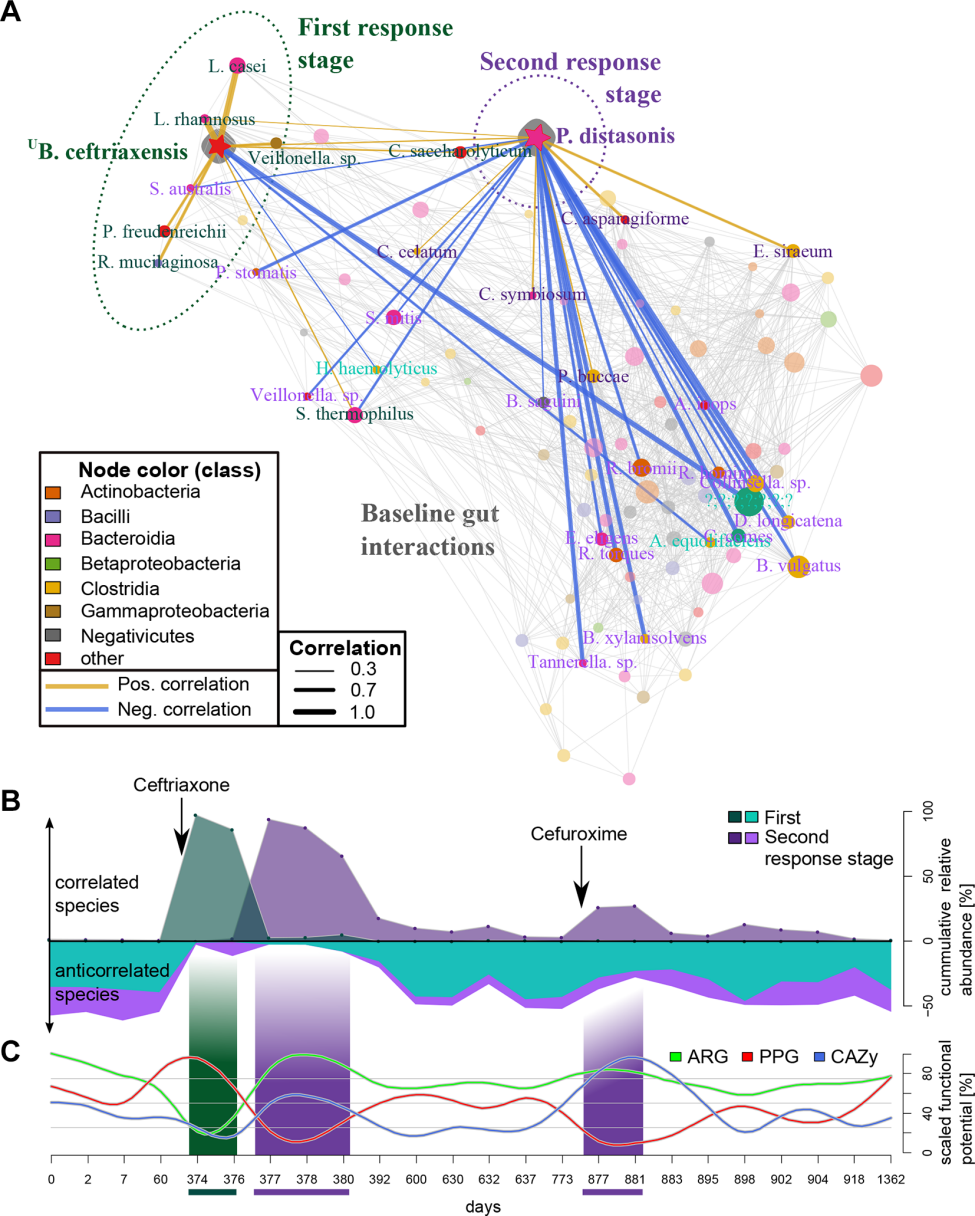
Microbial responses to antibiotics are reflected in their co-occurrences. (A) Patient-specific (subject HD.S1) species association network for 24 time points. The two monodominant species, ^U^ *Borkfalki ceftriaxensis* and *Parabacteroides distasonis*, are highlighted with their association networks. These networks differed substantially: ^U^*B. ceftriaxensis* was positively correlated to typical probiotic, lactose-fermenting bacteria. *P. distasonis* had only few positive correlations, with most associations being negative (18 associations), which could suggest a competitive or opportunistic role. These two interaction networks represent taxa associated to the first and second response stage, see (B). Nodes are coloured by their taxonomic class. Most associations are coloured grey, except those of ^U^ *B. ceftriaxensis* and *P. distasonis*, and only node names with associations to these are shown. Association line width and colour corresponds to Spearman correlations. Node size corresponds to average abundance of taxa in all HD.S1 samples. (B) The cumulative abundance of positive (above 0) and negative (below 0) taxa identified as part of the first and second response stage shows the dynamics of these subnetworks over time, coinciding with both antibiotic treatments. (C) Functional potential strongly varies between ecosystem successions (online supplementary tables 12 and 13). Carbohydrate active enzymes (CAZy) were enriched in the second response. Putative pathogenicity genes (PPG) were significantly increased in the first and significantly reduced during seconda response, while for antibiotic resistance genes (ARG) the opposite was true. CAZy, PPG and ARG are scaled by max/min abundance in HD.S1.

### ^U^ *B. ceftriaxensis* belongs to a new order of Firmicutes

To phylogenetically characterise ^U^ *B. ceftriaxensis,* we first analysed the 16S rRNA gene, the most widely used taxonomic marker. Empirical evidence suggests that 16S rRNA gene sequences are usually at least 95%, 92% and 89% conserved within the same genus, family and order, respectively.^17 25^ We confirmed that these thresholds apply to a set of 241 genomes representing all Firmicutes genera in the progenomes database,^26^ as well as four MAGs from human gut samples,^15^ that were selected for their similarity to ^U^ *B. ceftriaxensis* (online supplementary figure 5 and table 9). Our 16S rRNA gene analysis indicated that ^U^ *B. ceftriaxensis* represents a novel order, according to the above thresholds, as its nearest relative in the NCBI 16S database are the Firmicutes *Christensenella massiliensis* and *Catabacter hongkongensis* with 86% 16S sequence identity (NCBI blast). We further confirmed this using orthogonal approaches: although RDP classifier^27^ classifies the 16S rRNA to order Clostridiales at 100% confidence, it is not assigned to lower levels at the recommended 80% confidence, the closest assignment being to family Catabacteriaceae (53% confidence). The Microbial Genomes Atlas analysis^28^ indicated that this species represents a novel family or order (p=0.025, p=0.085, respectively). Our own within Firmicutes similarity-based analysis further confirmed its standing as a novel order (see online supplementary file 1).

To further investigate the phylogenetic placement of the new genome, we used the above set of 241 representative Firmicute genomes to construct maximum likelihood phylogenetic trees using either 40 conserved and universally present marker genes^29^ (figure 3) or the 16S rRNA gene sequence (online supplementary figure 6). Although most families and classes are monophyletic in this tree, some taxa are polyphyletic or clearly misassigned, reflecting previously discussed problems with the conventional Firmicutes phylogeny.^30^ Consequently, we propose a number of changes to the current taxonomy, as well as a split of the currently polyphyletic Ruminococcaceae family, creating two new families Erisaceae fam. nov. and Discordiaceae., fam. nov. (figure 3 online supplementary table 10, supplementary discussion)

^U^ *B. ceftriaxensis* is placed with high confidence into a clade together with three uncharacterised MGS, implying at least four distinct species, if not several genera (figure 3). They form a new family as the closest cultured relative to ^U^ *B. ceftriaxensis*, *Catabacter hongkongensis,* belongs to at least a separate family or order. Thus, to follow a standardised approach using empirical sequence similarity thresholds for classifying higher order clades, we propose that ^U^ *B. ceftriaxensis* belongs to the novel family ^U^Comantemaea fam. nov. and the novel order ^U^Comantemales ord. nov.

### ^U^ *B. ceftriaxensis* is a CMT

To get more insights into the ecology of ^U^ *B. ceftriaxensis*, we analysed the 4-year time series of subject HD.S1 (n=24). The ceftriaxone intervention caused extreme perturbation of the species composition (figure 1A, online supplementary figure 7), leading to two successive monodominance community states immediately after treatment. In both cases, the monodominant species is likely represented by a single strain (estimated to have <10 deviating fixed SNVs, online supplementary figure 4A,C). The first such state was identified 1 day after antibiotic treatment offset (day 374), with ^U^ *B. ceftriaxensis* representing 92% of all bacteria in this sample (corresponding to 2e11 cells/ml). This implies a 7742-fold and approximately 2000-fold increase in relative and absolute abundance, respectively, compared with preantibiotic samples (online supplementary figure 8). After this initial bloom, ^U^ *B. ceftriaxensis* was still monodominant 2 days later (60.6%, day 376), but then rapidly decreased to its baseline level, close to the theoretical species detection level (online supplementary figure 1). As this bloom coincided with an extremely reduced community diversity and absolute cell counts were reduced by 50% of the average (online supplementary figure 1), it appears to be opportunistic blooming into an ecosystem conceivably lacking colonisation resistance.

The extremely deprived community found after the ceftriaxone response was replaced with another monodominant community after only 3 days. This change was driven by the Bacteroidetes *Parabacteroides distasonis* (96.5% relative abundance on day 377), increasing 60-fold and 74-fold (relative and absolute abundance, respectively) relative to preantibiotic states. It represented the lowest observed richness and diversity in the whole time series. The *P. distasonis* monodominance lasted for at least 3 days, with 87% and 62% relative abundance on days 378 and 380, respectively, after which slow recovery was observed during the following weeks (online supplementary figure 9). *P. distasonis* was stable within the host HD.S1 during the whole observation period, represented by the same strain (online supplementary figure 10 for between individual analysis). Furthermore, the *P. distasonis* genome assembled from HD.S1 was almost completely horizontally covered in HD.S1 samples (median 98%), but only at less than 80% in almost all samples from other individuals (n=104), even when the vertical coverage was far deeper than some HD.S1 samples (online supplementary figure 2). This demonstrates the *P. distasonis* HD.S1 assembly being of high quality, as well as subject specific.

To generalise and validate our findings, we investigated species dynamics using a recently published metagenomics dataset from patients treated with a cocktail of three broad spectrum antibiotics (meropenem, vancomycin and gentamicin).^31^ Due to the dense sampling in this dataset we could replicate our results, by demonstrating a postantibiotic treatment monodominance (>60% relative abundance) occurring in 7 of 12 patients. Monodominant species varied, including *P. distasonis* as well as commensals such as *Bacteroides vulgatus*, *Bifidobacterium longum*, potential pathogens (*Escherichia coli*) and three species (206, 136 and 317) only identified in the mOTUs database.^13^ In addition,^U^ *B. ceftriaxensis* became more detectable after antibiotic treatment: it was detected in 5/12 and 11/12 patients before and 4 days postantibiotic treatment, respectively. Notably, it was highly abundant in 3 of 12 patients, detected at 13, 7 and 3.5% relative abundance in the latter samples. Its increase after non-beta-lactam antibiotics indicates a resistance extending beyond cephalosporine antibiotics, its likely presence at below detection thresholds in the healthy microbiome before antibiotic treatments in most patients and a general propensity to bloom into disturbed ecosystems.

To understand the impact of the apparent ^U^ *B. ceftriaxensis* resistance and monodominance along with the subsequent one of *P. distasonis* in our data set, we studied both short-term (<30 days) and long-term (up to 1362 days, the end of the time series) community responses in HD.S1.

### Short-term community response to ceftriaxone treatment

Of the three monitored interventions of subject HD.S1, the first, intravenous ceftriaxone treatment had the most severe effect on the microbiome. The microbial community was rebuilt from an almost barren ecosystem state to a complex, multispecies community. Two community rebuilding stages were observed, the first (days 374–376) and second (days 377–380; 877–881) response stages differing substantially in species compositions, dominant co-occurrence subnetworks and functional potential.

Rarefied species richness, diversity and evenness was reduced in postantibiotics samples (first and second stage, p=0.02, online supplementary figure 9) and the community was restructured at the class level: the first stage lead to an increase in *Clostridia*, whereas the second stage is characterised by a Bacteroidia increase (online supplementary figure 11). To investigate species interactions, we constructed a species co-occurrence over the whole time series, specific to subject HD.S1 (figure 4A). The first stage, being driven by the Firmicute ^U^ *B. ceftriaxensis*, lasted 2 days and was characterised by high abundance of a co-occurrence subnetwork that consisted mostly of typical small intestine and/or oral species (*Streptococcus thermophilus*, *Lactobacillus casei*, *Propionibacterium freudenreichii*, *Veillonella* sp., *Rothia mucilaginosa* and *Lactobacillus rhamnosus*) (figures 1A and 4A), most of which are typical lactose fermenters.^32^ In the second response stage, driven by *P. distasonis,* only few co-occurrences were observed, but instead co-exclusions to other species (n=17, figure 4A). After the intravenous ceftriaxone treatment, this second stage culminated in an extreme monodominance of *P. distasonis*. After an oral treatment with the second generation cephalosporin cefuroxime a year later (days 875–881), *P. distasonis* bloomed to 27% relative abundance and a similar co-exclusion of species emerged (figure 4B). This recurrent similarity between community states after cephalosporin treatment was also revealed by multivariate analysis on the taxonomic composition (online supplementary figure 7).

We further hypothesised that the community’s functional potential differs between the two stages, especially for functions related to resisting antibiotics (in form of antibiotic resistance genes (ARGs)), to invade and establish themselves in new environments (putative pathogenicity/invasion genes (PPG)) as well as the dominant carbohydrate active enzyme (CAZy) repertoire, as carbohydrate metabolism is reported to facilitate microbial recovery (see the Discussion section). A multidimensional analysis revealed a similar recurrent signal of the second stage response a year apart for CAZy, ARG and PPG composition (online supplementary figure 12). All three functional signatures varied significantly between stages, with the general pattern being an increase in CAZy and ARG potential in the second stage, while the first stage was enriched for PPG genes (figure 4C, online supplement result). CAZymes categories specific to food-derived (plants, animals) carbohydrates were increased during both second stages, especially after cefuroxime treatment, in contrast to decreased CAZYmes specific to microbial substrates (bacterial, fungal) (online supplementary figure 13), which could be interpreted as higher trophic levels being decreased during initial community rebuilding.

### Long-term ecosystem changes after antibiotic treatment

**Figure 5:**
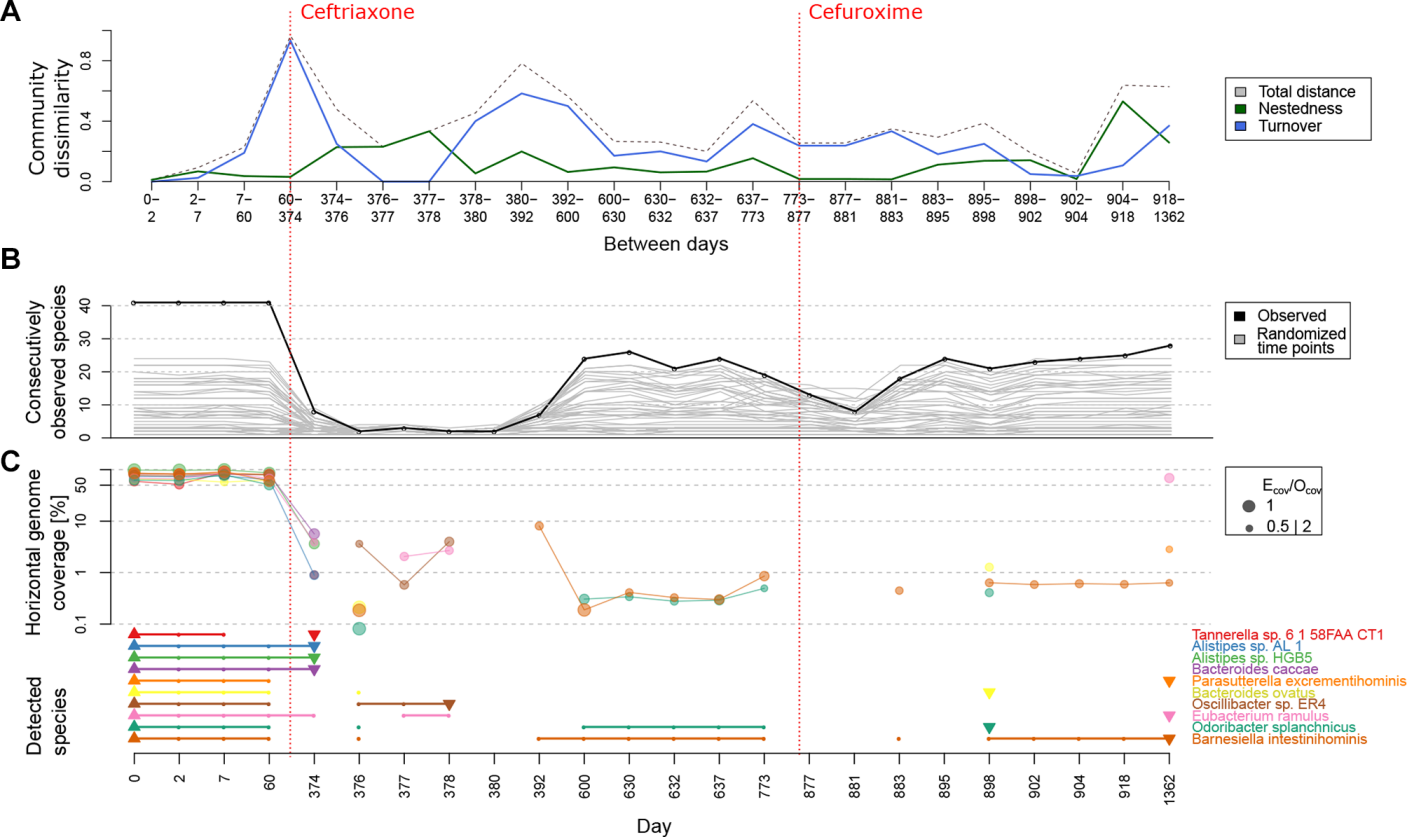
Species loss after ceftriaxone treatment in subject HD.S1. (A) Comparing components of community variation between time ordered samples revealed a strong community turnover followed by a peak in species loss/gain (ie, nestedness) after ceftriaxone treatment. We hypothesised that this led to permanent loss of certain species in patient HD.S1. (B) Forty-one species could be detected in all four preantibiotic time points, but 10 of these were absent from postantibiotic samples. (C) We further tested the presence of these 10 species with a more targeted approach, mapping metagenomic reads against their reference genomes. Mappings were filtered to include only a ratio of expected (E_cov_) and observed (O_cov_) horizontal coverage between 0.5 and 2. The occurrence is noted in the lower part of the plot, marking the first detection of a species with an upward triangle and the last observation with a downward triangle. The analysis shows that most species were present at high horizontal coverage before the intervention and generally not observed after the intervention, or with such a low horizontal coverage that observation of their marker genes was statistically unlikely, that is, they are not detectable by marker based approaches. The second antibiotic treatment seems to reduce the surviving species from the initial set. Note that the range of <10% genome coverage could as well represent mobile elements or highly conserved genes being preserved in other species and is therefore the threshold applied to confirm species detection.

Monodominance is likely a fragile state of adult gut microbiomes, as lower diversity is generally associated with decreased resilience in most ecosystems.^33^ Further disruptions might lead to detrimental ecosystem changes^34^ including catastrophic regime shifts. While we did not observe a catastrophic regime shift, we could detect long-lasting changes after ceftriaxone treatment, comprising an apparent loss of nine species and a shift in species dominance patterns in subject HD.S1. We discovered a strong species turnover after ceftriaxone treatment (figure 5A), indicating an almost complete replacement of species in adjacent time points. Intriguingly, Betaproteobacteria were almost undetectable after the initial antibiotic treatment and for the remainder of the time series, although often being associated with diarrhoea associated blooms following antibiotics.^10^ The Bacteroidetes *P. distasonis* was the only taxon significantly increased in relative abundance after the ceftriaxone treatment in all subsequent time points (p=0.009, mean relative abundance 1.3%±0.3% pretreatment vs 20%±28%, online supplementary figure 1). In contrast, 26 species were significantly decreased in relative abundance (online supplementary table 11 and figure 14). Of these, 10 species could only be detected in all four samples before ceftriaxone treatment, but in none of the 20 samples afterwards. To investigate species losses more specifically, we traced the 41 species consistently present in all preantibiotic samples: this analysis showed that 75% were still present in subject HD.S1 and in some post-ceftriaxone samples. However, 10 species could no longer be detected (figure 5B). This pattern is highly unlikely to occur by random chance: in 100 000 reshuffled time labels, this never occurred for several species, and even one species loss only happened in 0.094% of the reshuffled trials.

Because it is difficult to differentiate complete loss of a species from depletion to very low abundance, we calculated for these 10 species the observed horizontal genome coverage (O_cov_), filtering for O_cov_ consistent with expected coverage (see online supplementary file 1). Validating our marker-gene based approach, the median O_cov_ before ceftriaxone was 79% (figure 5C). Detecting even genomic fragments of these species was difficult in post-ceftriaxone samples: four species could not be detected at all after day 374 and O_cov_ was extremely low among the remaining six species (0.6% median). Only *Eubacterium ramulus* was detected at >10% O_cov_ at the last sampling point, 2.7 years after the initial antibiotic treatment and SNV analysis indicates this being the same strain; thus nine species appear to be lost to this ecosystem. Interestingly, there was a consistent low O_cov_ for four species on the day after antibiotic treatment (day 374) that could either be washout of dead cells or indicate that these species lack essential symbiotic species^35^ and could therefore no longer maintain themselves after community restructuring. Following the second antibiotic treatment, there was a consistent drop in O_cov_ among detected species as well as species detected via marker genes (figure 5B, C), suggesting that the same species suffer from cephalosporin treatments deterministically, having not attained an effective resistance mechanism.

Together, it appears that a regime shift^36^ occurred after the ceftriaxone treatment, with key indicators of an altered gut community being the considerable, long-lasting increase of *P. distasonis* relative abundance as well as the likely loss of several species.

## Discussion

We have identified a novel, endogenous, conditionally monodominant taxon (CMT) in the human gut microbiome, ^U^ *B. ceftriaxensis*, which is associated with the human host over long time periods. It is prevalent in one-third of public gut samples, but in general low abundant. Its discovery was enabled through its high abundance in postantibiotic stool samples and subsequent, reference-free genome binning. Additional mate-pair sequencing allowed the reconstruction of a high-quality genome from metagenomics data, as all culturing attempts were unsuccessful. Phylogenetically, it belongs to the phylum Firmicutes and class Clostridia, being the first representative of ^U^Comantemales, ord. nov.

^U^ *B. ceftriaxensis* and *P. distasonis* were driving two observed monodominance community states that peaked at 92% and 95% relative abundance, respectively. These states could be common in the gut, given that during their lifetime, many individuals are frequently subjected to gut microbiome perturbations resulting, for example, from medication, disease or short-term digestion disturbances. Such monodominances could have remained unnoticed so far, either due to technical issues (discussed in the online supplementary file 1) or because they did not exhibit phenotypic effects during monodominant phases, like subject HD.S1. Antibiotic interventions are often, but not consistently, reported to decrease gut microbial diversity during and after treatments, followed by a recovery of the microbial community within weeks^37^ or months.^38^ The intravenous antibiotic treatment of HD.S1 shifted the postantibiotic microbiome composition significantly: several species appeared to have been simultaneously lost after treatment, compared with none at any other time point. Our simulations imply that such a non-detection over 20 time points and 1392 days due to random abundance fluctuations is highly unlikely. The loss of species after antibiotic treatments has only been reported recently on much shorter time scales and using only marker genes.^31^ Importantly, such shifted communities might induce long-term consequences that are not immediately noticeable like weight change,^1^ diabetic^39^ or maybe even Parkinson’s disease predisposition.^40^

Short-term monodominant states can be essential parts of successions^36^ towards the restoration of a complex ecosystems.^41^ Here, through hysteresis,^42^ ^U^ *B. ceftriaxensis* could have changed the community type in the longterm to what appears to be a healthy, but shifted, community state. For example, although no probiotics were consumed, most species co-occurring with ^U^ *B. ceftriaxensis* were probiotic species, frequently used to treat antibiotic associated diarrhoea (AAD).^43 44^ In an independent cohort,^31^ we discovered its enrichment following antibiotics treatments. This shows the potential importance of ^U^ *B. ceftriaxensis* as a keystone species in rebuilding the gut community and as a probiotic candidate in future studies. The metabolism of ^U^ *B. ceftriaxensis* fits to this role, putatively fermenting a wide spectrum of simple carbohydrate sources to multiple short-chain fatty acids (figure 1C) that may suppress AAD.^45 46^

*P. distasonis*, the main driver of the second postantibiotics response stage, has been associated to faster microbiome recovery after antibiotic interventions.^46^ Bioavailable carbohydrates are important in the microbial recovery process^45 46^ and the second response stage was increased in total carbohydrate active enzymes (figure 5C). This type of community is also observed after another antibiotic treatment 1 year later and appears to be part of a semi-deterministic community response to antibiotics. Although earlier studies indicated that the microbial antibiotic response differs between individuals,^37^ studies with larger cohorts found that groups of patients can have similar recovery patterns, dependent on the initial composition.^46 47^ Therefore, the characterisation of the personal microbial antibiotic response should be an important goal of personalised medicine to enable a guided therapy. This might significantly increase effectiveness of treatments, prevent AAD and avoid the negative long-term consequences of species loss in the microbiome.

In summary, our analysis revealed a by-far underestimated dynamic nature of the human gut microbiome through the successive, extreme blooming of two commensal species after antibiotics treatment. The fast community restructuring allowed the characterisation of an otherwise low abundant species. Short-interval sampling after perturbations of the gut microbiome would further elucidate the role of conditionally monodominant gut species, such as ^U^ *B. ceftriaxensis,* in resilience to perturbations, with prospective usage as indicators for disturbed microbial states.

### Description of novel taxa

#### Description of Erisaceae fam. nov

Gr. fem. Noun, derived from Gr. Eris, Greek Goddess of discord and strife, since Ruminococcaceae family is phylogenetically separated by this taxon. Formerly of family Ruminococcaceae, it includes genera *Ruminiclostridium*, *Acetivibrio*, *Pseudobacteroides* and *Clostridium* and the following species: *Clostridium cellulyticum*, *Clostridium josui*, *Pseudobacteroides cellulosolvens*, *Ruminiclostridium thermocellum* and *Acetivibrio cellulyticus*. This nomenclature is supported by maximum likelihood phylogeny based on 40 conserved universal marker genes as well as 16S analysis.

#### Description of Discordiaceae fam. nov

M.L. fem. Noun, derived from Lt. Discordia, Latin goddess of discord, since Ruminococcaceae family is phylogenetically separated by this taxon. The family contains one named genus, *Maegeeibacillus*, and one named species, *Maegeeibacillus indolicus*, supported by maximum likelihood phylogeny based on 40 conserved universal marker genes as well as 16S analysis. Further, it may contain two species of uncertain taxonomic origin: *Ruminococcaceae bacterium* AB4001 and *Ruminococcaceae bacterium* AE2021 that fall within this family based on 40 conserved universal marker gene, but this is not supported by 16S analysis.

#### Description of ^U^Comantemales ord. nov

Comatemales (Co. mān.te.ma.les. N.L. verb comantem, meaning blooming.). The order is represented by four genomes, ^U^ *B. ceftriaxensis*, ‘CAG727’, ‘CAG314’ and ‘CAG349’, all of which are metagenomically assembled genomes without a cultured representative, but are available in public databases. The closest relative is *Catabacter hongkongensis*, which based on 16S rRNA gene similarity is of a different order.

#### Description of ^U^Comantemaea fam. nov

Comatemaea (Co.mān.te.maea. N.L. verb comantem, meaning blooming.). The family is represented by four genomes, ^U^ *B. ceftriaxensis*, ‘CAG727’, ‘CAG314’ and ‘CAG349’, all of which are metagenomically assembled genome without a cultured sample but are available in public databases.

#### Description of ^U^ *Borkfalki* gen. nov

*Borkfalki* (Bork.fālk.í. (abbreviation) acknowledging the persons where the type strain was first described. The guanine-cytosine content of the genome of the type strain HDS1380 is 51.7%. The type species is ^U^ *B.ceftriaxensis*, which is a member of order Clostridiales, phylum Firmicutes, according to 16S rRNA gene analysis as well as analysis of 40 phylogenetically conserved marker genes.

#### Description of ^U^ *B. ceftriaxensis* sp. nov

*^U^B. ceftriaxensis* (cef.tri.a.xen.sis. N.L. Arbitrary name. N.L. fem. adj. ceftriax- (abbreviation), ceftriaxone, the antibiotic on which proliferation of this species was observed; N.L. (abbreviation) fem. noun ensis, derived from Gr. Nemesis, Greek goddess of retribution, as in antagonist). The bacterium is rod shaped and 1.5×0.8 µm in dimension. It is associated with the human microbiome, preferentially gut, but was so far not detected in other environments (animal guts, environmental samples). Bioinformatic annotation indicates that it is spore-forming anaerobe with fermentative metabolism. It can metabolise a wide range of mono and oligosaccharides (see online supplementary methods). Likely metabolic products include ethanol, lactate and acetate. Considering the genomic context, it is likely that it metabolises 1,2 propanediol to propanol and propionate, although the gene coding for the enzyme to produce the latter was not annotated. It can likely metabolise a wide range of monosaccharides and disaccharides (alpha-L-fucoside, alpha-L-rhamnoside, alpha-trehalose, beta-glucoside, cellobiose, fructan, galactoside, L-arabinose, maltose, mannosides and mannose, sucrose and xylan). The type strain’s genome, HDS1380, was obtained from the metagenome of a patient being recently treated with the antibiotic ceftriaxone.

10.1136/gutjnl-2018-317715.supp3 Supplementary file 3

10.1136/gutjnl-2018-317715.supp5 Supplementary file 5

10.1136/gutjnl-2018-317715.supp6 Supplementary file 6

10.1136/gutjnl-2018-317715.supp8 Supplementary file 8

10.1136/gutjnl-2018-317715.supp9 Supplementary file 9

10.1136/gutjnl-2018-317715.supp10 Supplementary file 10
