# Supplementary material for: Antibiotics-induced monodominance of a novel gut bacterial order
Source: Gut. 2019 Jan 18;68(10):1781–90. doi: 10.1136/gutjnl-2018-317715 (PMC6839795; doi:10.1136/gutjnl-2018-317715)
Supplement: Supplementary file 1 [file gutjnl-2018-317715supp001.pdf]

Materials & Methods

Patient cohort

All samples from participants in the cohort are publicly available (see database below), with the exception of samples listed in Suppl. Table 8. These samples were obtained from a Heidelberg centered cohort within the my.microbes project (my.microbes.eu), as described in greater detail in [1]. The study was approved by the EMBL Bioethics Internal Advisory Board and adheres to the WMA Declaration of Helsinki. Fecal samples were collected and conserved under anaerobic conditions and kept for long term storage at -80°C, after a brief short-term storage period at -20°C. Patient HD.S1 was undergoing bowel cleansing on day 630 for a routine colonoscopy unrelated to the study.

Furthermore, patient HD.S1 was treated twice with antibiotics, first on days 370-374 with 2g/day intravenous ceftriaxone. The last injection with ceftriaxone occurred approximately ~12-24 hours before HD.S1.374 fecal sample collection. The patient was being treated for a bacterial infection after a kidney stone operation. We assume that at the time of collection of sample “HD.S1.374”, there were still significant concentrations of ceftriaxone (12-25% of treatment dose) in the patient’s urine and feces. This assumption is based on the elimination half-life of ceftriaxone being 5.8-8.7 hrs, and 12 hrs for patients with renal impairment (https://www.drugs.com/pro/ceftriaxone.html, subject HD.S1 had a kidney stone operation). The second antibiotic treatment lasted six days, starting on day 875, with CefuHEXAL® 500 mg, 2 oral tablets per day.

Sequencing of fecal samples

Genomic DNA was extracted from frozen fecal samples as previously described [1] using the GNOME© DNA Isolation Kit (MP Biomedicals). Library generation and random shotgun sequencing of fecal samples was carried out on the Illumina HiSeq 2000/2500 (Illumina, San Diego, CA, USA) platform. All samples were paired-end sequenced with 100 bp read lengths at the Genomics Core Facility, European Molecular Biology Laboratory, Heidelberg, to an approximated sequencing depth of 5 Gbp per sample. Further, sample HD.S1.377 was sequenced to greater depth and with longer reads using the Illumina MiSeq platform, obtaining 2x250 bp paired end reads. In addition to this, sample HD.S1.377 was sequenced with mate pair sequencing using Illumina Nextera Mate Pair Library Prep Kit to assist genomic assemblies of ^{U}*B. ceftriaxensis* and *P. distasonis*.

Attempted culturing of ^{U}*B. ceftriaxensis*

The RAST annotation of ^{U}*B. ceftriaxensis* served as basis to create a metabolic profile to guide the selection of carbohydrates for isolating this species (Suppl. Table 1). The faecal samples (~0.25g) at day 375 containing ~60% and day 374 containing ~90% of the novel species were dissolved in 5ml PBS/20% Glycerol under anaerobic conditions. The basic media was CPYC (CP medium [2] with an addition of Yeast extract 2.5g/l Casitone 10g/l). The additional carbohydrates were added to 25mM end concentration. The carbohydrates used were either Rhamnose, Trehalose, Raffinose, Ribose, Lactose, Sucrose, Maltose, Glycerol, Xylose, Xylan, Mannose, Galactose and a mix of all carbohydrates together. Apart from liquid cultures solid media was also used to attempt isolation of the species. The plates contained BHI or CPYC supplemented with the above mentioned carbon sources. All bottles were inoculated with ~100ul of fecal sample/PBS/Glycerol. Plates were streaked and incubated at 37°C.

After incubation the liquid cultures were inoculated in new bottles with their carbohydrate and anaerobically plated on BHI with their selective carbon for isolation. This process was repeated until pure cultures or single colonies were apparent. Additionally, 16S specific primers based on the ^{U}*B. ceftriaxensis* 16S were designed (Suppl. Table 2) with NCBI Primer Blast[1] for screening the obtained enrichments. Using these primers on the culture media, we were able to detect the species in ribose- and rhamnose-supplemented media. Subsequently, the 16S rRNA gene of the isolates was sequenced using the Sanger method to confirm the isolate's identity. However we did not retrieve identical 16S sequences to TEC-2, instead recovering (based on 16S similarity >= 98%) *Eggerthella lenta*, *Tetragenococcus koreensis*, *Lactobacillus rhamnosus* and *Intestinibacter bartlettii*.

Fluorescence *in situ* hybridization to detect ^{U}*B. ceftriaxensis* in fecal samples

Oligonucleotide probes specific for ^{U}*B. ceftriaxensis* 16S rRNA were designed using the probe design tool implemented in ARB (SILVA Release 132 [3]). Specific hybridization conditions were predicted *in silico* using mathFISH (http://mathfish.cee.wisc.edu) and were experimentally validated by CLONE-FISH against the target region heterologously transcribed in *Escherichia coli* as detailed in [4]. Probe TEC483 (5'-CGA GGC TTG CTA TTG GGA TAC CG-3') was chosen for FISH experiments and was synthesized double Cy3 labelled at 5'- and 3'- ends (Sigma-Aldrich, Steinheim, Germany). Fecal samples were suspended in 1% paraformaldehyde: dPBS, fixed

[1] https://www.ncbi.nlm.nih.gov/tools/primer-blast/index.cgi?LINK_LOC=BlastHome

overnight at 4°C and stored in 50% EtOH:dPBS at -20°C until further processing. Cell preparations were heat fixed on glass slides and hybridized for 2 hours in 30% formamide hybridization buffer as described in [4].

Bacterial cell counting using flow cytometry

To analyze the density of bacteria cells, 100 µL of frozen stools were suspended in 0.9% saline solution and diluted 1:100. Samples were homogenized by vortexing with sterile Rattler™ Platting Beads (Zymo Research). To remove all remaining big particles, the suspension was filtrated by gravity flow through a 40 µm Cell Strainer (Falcon®). Samples were further diluted to 1:1000 000 and stained using a modified protocol for Bacteria Counting Kit (Invitrogen™). Briefly, 1 µL of SYTO® BC bacteria stain was added to 1.0 mL of diluted cells and incubated in darkness at 37°C, for 30 min. Bacteria were centrifuged at 10,000 rpm for 15 min and the pellet was re-suspended in 500 µL of 4% paraformaldehyde and incubated for 30 min at RT. Fixed cells were washed and re-suspended in 1 mL of saline solution. 10 µL of the Bacteria Counting Kit (Invitrogen™) microsphere standard suspension were added and cells were analysed using LSRFortessa™ (BD) with excitation 488 nm and emission at 530/30 nm. The number of bacterial cells per number of microspheres was used to calculate the density of bacteria in 1 mL of stool sample in triplicate. The mean of the triplicate value was then used to determine cell density per sample.

Assessing of beta-lactamase activity and antibiotic resistance

Both beta-lactamase genes found in ^{U}*B. ceftriaxensis* were placed under the control of the amp promoter into the pMK-RQ backbone (kanR). The CTX-M 15 beta-lactamase encoded on the pEC499 plasmid was used as a positive control (Woodford et al. 2009). All constructs were ordered ready to use from GeneArt AG and transformed into *E. coli* (strain DH5α) for constitutive beta-lactamase expression.

Antibiotic resistance was assessed on LB-amp and LB-ceftriaxone plates at antibiotic concentrations of 100 mg/l and 159 mg/l, respectively. Beta-lactamase activity was tested using nitrocefin disks (Sigma) that change color from yellow to red when beta-lactamase activity is detected.

Read filtering

Reads obtained from the shotgun metagenomic sequencing of the 129 metagenomic samples from the focused dataset were quality-filtered by removing reads shorter than 70% of the maximum expected read length (100 bp, 250 bp for miSeq data, 150 bp for mate pair reads),

with an observed accumulated error >2 or an estimated accumulated error >2.5 with a probability of ≥0.01 [5], or >1 ambiguous position. Reads were trimmed if base quality dropped below 20 in a window of 15 bases at the 3' end, or if the accumulated error exceeded 2 using the sdm read filtering software [6]. Human reads were removed from the metagenomic data by classifying raw reads with kraken [7] against a custom database containing solely the human reference genome. Unclassified reads were further used in downstream analysis.

Estimating abundance of genomes

To estimate if a genome was present, we first mapped reads from all samples against the reference genomes obtained from NCBI with the same mapping procedure and depth estimation as described above. From these mappings we estimated the horizontal coverage across the genome and the total number of reads mapping to a reference. Because these genomes could be extremely low abundant in the timeseries data, often having a horizontal coverage < 25%, we instead used the following formula to estimate the expected coverage (E_{cov}), following [8], with G_L being genome length, n the number of mapped reads and R_L being the read length:

$$E_{cov} = 1 - G_L * exp\left(\frac{-n * R_L}{G_L}\right)$$

Since the mapping was prone to attracting short reads to conserved or mobile genes, that is genomic regions with extreme coverage, we used a standard MD filter [9] to remove obviously overrepresented genomic regions prior to calculating the expected coverage,.
Comparing the observed coverage (O_{cov}) and expected horizontal coverage, we only accepted genomes as being present, if $0.5 < E_{cov} / O_{cov} < 2$ (arbitrarily chosen). The ratio of E_{cov} / O_{cov} as well as O_{cov} is reported in (Fig. 5)

Metagenomic assembly and gene catalog

Only sdm [6] filtered paired reads were used in the assembly, with the same read filtering parameters as described above. In total 10,865,401,856 reads were assembled using Spades 3.7-0 (development version obtained from the authors) [10] in metagenomic mode with the parameters "--only-assembler -m 500 --meta -k 21,33,67,111,127". The samples were assembled separately for each biological sample, to decrease the chances for chimeric contigs, but technical replicates were pooled. The assembled contig had an average N50 of 14,041 bp, scaffold average N50 was 17,128 bp and a total size of 31,782 Mbp. Using prodigal [11] in

metagenomic mode, 38,943,896 genes were predicted on the contigs. Only genes with >100 bp length were selected and split into 15,558,517 complete genes and 47,076,247 incomplete genes. The average complete gene length was 897 ± 65 bp.

In a first step, the complete genes were clustered at 95% nucleotide identity, a commonly used cutoff in constructing gene catalogues (Sunagawa, Coelho, Chaffron, Kultima, Labadie, Salazar, et al. 2015). For sequence clustering CD-HIT v4.6.1 [12] was used in est mode, employing parameters adapted to full length genes: “-n 9 -G 1 -aS 0.95 -aL 0.6 -d 0 -c 0.95 -g 0”. This resulted in 3,257,016 clustered full length genes, onto which the incomplete genes were mapped with bowtie2 [13]. Incomplete genes mapping with at least 95% nucleotide identity were directly integrated into the initial clustering of complete genes. The remaining sequences were clustered as before with CD-HIT, but changing alignment length parameters to “-aL 0.3 -aS 0.8” to account for incomplete genes, resulting in 2,466,595 additional gene clusters. Additionally, genes belonging to the 40 conserved marker genes were clustered separately, using clustering identity thresholds as described in [14]. Merging marker gene clusters, incomplete and complete clustered genes resulted in the gene catalogue, with a total of 5,790,967 non-redundant genes at 95% nucleotide identity cut-off. The gene catalogue nucleotide and amino acid sequences are available at *http://vm-lux.embl.de/~hildebra/Bcef_GC*.

Estimating species abundances in metagenomes

To estimate the species abundance per sample and at the same time also infer the species not in our database, we used the 40 single copy ubiquitously present marker genes (MGs) that can be predicted using specI [14] in the gene catalog. Since hidden markov models trained on the tree of life are used for this prediction, we can assume that the MGs of unknown classes, families, genera will be detected in this approach. From this set of marker genes, we used a similarity based approach to identify species within these, mapping all predicted MGs with lambda 1.9.3 [15] onto MGs clustered to specI’s in the proGenomes database [16] and using a MG specific similarity cutoff [14]. Hits were immediately accepted, if a metagenomic MG was hitting a single proGenomes specI at the required similarity threshold. However, since several metagenomic MGs had valid hits to multiple proGenomes MGs, we supplemented the identification of a species using a coabundance approach, in concept similar to [17]. Briefly, first for established specI’s we calculated the average profile across all MGs. If the MG showed similarity to a given specI, i.e. was a candidate, the remaining MGs that were not uniquely

assigned to a specI were correlated to existing specI profiles. Here we also used species and genus taxonomic assignments, to allow for mismatches in nucleotide sequence. MG's with a spearman correlation coefficient > 0.9 to a candidate specI were immediately added to a given specI.

Additionally, at different phases in the clustering algorithm, within each specI, MGs were checked to correlate with the average profile (spearman) < 0.9, or were removed as false positive assignments and iteratively tried to be added to different specI's, as described above.

Mapping reads against references

To estimate the abundance of genes in each assembly, the reads from a sample were mapped against the assembly of these. Furthermore, in specific cases also all reads were mapped against reference genomes (to estimate genome presence) or reference genome + decoy set (see below) to map reads against a reference genome in order to call genetic variants. For these use cases, all unfiltered reads were used in the mapping, irrespective of quality. This was important as we were interested in very low abundant species and quality filtered reads were in this project important to ensure a clean assembly; if reads were mapping at a good quality against this assembly it was assumed that their quality was sufficient. To ensure good matches, bowtie2 [13] was used with the options "--no-unal --end-to-end --score-min L,-0.6,-0.6" and additionally "-X 10000" in the case of mate pair libraries. The resulting bam files were sorted, duplicates removed and indexed using samtools 1.3.1 [18]. From these depth profiles were created using bedtools which were translated with a custom C++ program "rdCov" (https://github.com/hildebra/rdCover) into average coverage in a 50 bp window, per contig or per gene predicted on each contig.

Functional annotations of protein sequences

Translated amino acid sequences of either complete genomes, gene catalogs or contigs were predicted with prodigal [11] in normal mode, or metagenomic mode for the latter two cases. These amino acid sequences were mapped to functional reference databases using diamond [19] in blastp mode using options "-k 5 -e 1e-5 –sensitive". The reported mapping were further filtered using customized Perl scripts, with filtering parameters set according on simulations reported previously (Bahram et al. 2018). In general, we required for each database that hits had >25% AA identity, >30% gene coverage, >60 bit score, >60 AA acid alignment length and an e-value <1e-7. The highest scoring hit was then accepted.

The following databases were used for functional assignments: Cazyme genes (http://www.cazy.org, accessed 22.11.2015)[20], KEGG [21], antibiotic resistance genes

(ARG's) curated in CARD [22] and eggNOG 4.5 database of functional orthologs [23] genes. For ARG, only hits scoring better than the CARD curated cutoffs [22] were accepted. Further, we created from PATRIC [24] a virulence factor specific database. The gene, protein and genome sequences of the PATRIC database, along with the sets of 'specialty genes' assignments made by its curators, was downloaded in November, 2017. From this dataset was parsed assignments to the PATRIC virulence factor classifications, with genes not assigned to a virulence factor considered a true negative given the denseness of the database. The VF gene symbols and classes together with the sequences were considered as input data for further steps annotating metagenome-derived entities with virulence factor information.

To facilitate interpretation of the results, the relative abundance of CAZyme genes were summed based on the substrates for each gene family. Substrate utilization information for CAZyme families was obtained from [25,26] as well as CAZypedia (http://www.cazypedia.org/). SEED functional annotations were obtained from the web interface of RAST [24]. Based on the KEGG Ortholog (KO) abundance matrices we further calculated KEGG and GMM (Darzi et al. 2016) module abundances using a custom C++ implementation available on www.github.com/hildebra/Rarefaction, similar to the protocol described in [27].

Functional abundance matrices were normalized by total number of reads used in mapping. This normalization considers differences in library size and includes the fraction of unmapped (that is functionally unclassified) reads. Replication times of assembled genomes were estimated using [28].

Genome binning

Genomes were de novo binned for ^{U}*B. ceftriaxensis* and *P. distasonis*. This was based on binning samples HD.S1.374 and HD.S1.377, respectively, with MaxBin [29] and a data set wide binning via Canopy clustering [17]. MaxBin was run with default options, except for the changed option "-min_contig_length 400".

Canopy clustering was run with options to optimize for rare, highly abundant species: "-p TC --die_on_kill --stop_criteria 250000 --cag_filter_min_sample_obs 5 --cag_filter_max_top3_sample_contribution 1 --filter_max_top3_sample_contribution 1" on a gene abundance matrix that was normalized by sum using rtk [30]. The gene bins of both binning methods were combined and the abundance profiles of the respective genes further analyzed in R.

For each species, a pearson correlation matrix for the candidate gene profiles were created, and the inverse of correlation coefficient was used to hierarchically cluster these (R function

hclust, method = complete). The hierachical cluster was iteratively subdivided, until all 40 marker genes were in the same cluster (illustrated in Suppl. Fig. 16 for ^{U}*B. ceftriaxensis*). Based on this clustering, the metagenomic assembly was selected combining most target genes in the fewest scaffolds. In a last step the scaffolds based on HiSeq paired end reads were further scaffolded with mate pair reads (insert size = 5000) from sample HD.S1.377. The mate pair reads were mapped onto the selected species contigs using bowtie2 [13]. Based on these mappings, scaffolds were calculated using BESST [31] using default parameters with the exception of "-z 5000". In the case of ^{U}*B. ceftriaxensis*, this decreased the number of scaffolds in the primary Spades assembly from 20 to 8 scaffolds. Genome completeness was estimated using CheckM [32] and MiGA [33].

Phylogeny of ^{U}*B. ceftriaxensis*

From the proGenomes database [16], we selected a random subset representing each Firmicutes family within the database with at least one genome, but where available representing each genera in the database. Further, this tree was supplemented in August 2017 with marker gene based searches in NCBI for complete genomes that had a high similarity to the ^{U}*B. Ceftriaxensis* marker genes. Further, we also included 3 genomes based on metagenomic binning [17] (CAGs, co-abundance groups), because the marker genes of ^{U}*B. ceftriaxensis* showed similarities to these genomes, when searching NCBI NR.

For all genomes in this database (n=242) two sets of marker genes were extracted. First the 16S rRNA gene was obtained from each genome with RNAmmer [34], which reduced the species set to 236 genomes since the CAG's did not have a detectable 16S rRNA gene and 3 further genomes no reference 16S gene could be found. The 16S sequences were aligned with Clustal Omega [35] with default options and inspected by hand. Subsequently a maximum likelihood tree was constructed with IQ-TREE[36] using ModelFinder [37] to find the best substitution model and calculating 100 bootstrapped trees and using booster [38] to calculate the bootstrap support.

Second, a phylogenetic tree was constructed from 40 single copy universally present marker genes. These were obtained with fetchMG [14]. The genes were translated into amino acid sequences and aligned separately for each of the 40 orthologues gene groups with Clustal Omega [35] (default options). Subsequently, the multiple sequence alignments were quality controlled with trimal (options -keepheader -ignorestopcodon -gt 0.1 -cons 60) and merged. The phylogenetic tree was reconstructed from this amino acid alignment of 40 orthologues from 242 genomes using IQ-TREE, as described above to obtain phylogenetic trees with bootstrap

support. Both maximum likelihood trees were visualized using iTOL [39]. The nucleotide and amino acid identity between sequences was calculated from either multiple sequence alignment, using custom Perl scripts (included in MATAFILER).

Global distribution ^{U}*B. ceftriaxensis*

The abundance of ^{U}*B. ceftriaxensis* was measured in our global (n=3,692) sample set, using the mOTU approach [40] and including custom marker genes derived from the genome assembly. Our reference database contained 70 internal German samples [1,41], 368+156 Chinese cancer samples [42,43], 1209 American HMP samples [44], 194 German colorectal cancer samples [45], 387 Danish samples [27], 145 Swedish Samples [46]. This was complemented by non-human samples, consisting of 295 pig gut, 191 mouse gut, 124 cat gut and 129 dog gut [47,48] samples. Further, we included environmental metagenomes from 243 Tara Oceans samples [49] as well as 189 global topsoil samples [50]. From the non-normalized mOTU abundance tables, we estimated what percentage of inserts was representative of ^{U}*B. ceftriaxensis*.

For the discovery dataset, samples from the above mentioned dataset were included, with the addition from twelve antibiotic treated human gut microbial samples [51]. We report for this focused dataset the 129 samples with their accession and sample IDs in Suppl. Table 8.

Decoy mapping of reads

In human variant calling, the reference genome (e.g. hs37d5) contains often a so-called decoy contigs, that cover recurrently (but rarely) assembled regions that are not part of the primary assembly of the human genome [9]. In order to decrease the rate of false positive read mappings in the much more complex metagenome with many species that are sometimes closely related, we used a similar principle. However, instead of adding new reference genomes we took the inverse approach relying on our metagenomic assembly, which should contain the best matching references to any reads from a given sample (see section metagenomic assembly). From this assembly contigs similar to the mapping target were first removed. To do this, the metagenomic assembly was mapped on the target sequences using blat [52] with the following parameters: -t=dna -q=dna -minIdentity=95 -minScore=100. These identified matching contigs were removed from the assembly, and the remaining assembled contigs and the reference genome were used as new database to map the metagenomic reads against. In our parameter tests, this procedure increased average mapping scores at variant calls (data not

shown) and was therefore used for all SNP calls. The script to create decoy mappings is available in the MATAFILER pipeline.

Estimating dominant strain and its genetic variation

First reads from a given sample were mapped against the reference genome, using the above described decoy mapping procedure. From these, nucleotide variants were called using freebayes ver. 1.1.0 [53] with the following options -m 30 -q 30 -u -i -C 1 -F 0.1 -k -X --pooled-continuous --report-monomorphic --min-repeat-entropy 1 --use-best-n-alleles 2 -G 1. Freebayes is a variant caller that corrects for misalignments using a local realignment among other features, adapted to SNV calling in highly heterologous cancer genomes. The output vcf file was filtered with a custom Perl script (vcf2cons.pl, available as part of the MATAFILER pipeline), that created a consensus sequence from the reference allele, unless the alternative allele frequencies was >0.501. The minimum coverage was set to 2 alleles. The 0.501 threshold serves to avoid introducing a reference bias in the consensus sequence and to filter reads assignments at a depth of 2, that have a 50% allele frequency.

Further, using the 50% allele frequency cutoff circumvents the possibility of several strains being present in the same host, by only estimating the genome of this strain. If a second strain would in another time point arise to become the dominant strain, this would be immediately inferable from the within host diversity and flagged as a strain exchange (which happened in two hosts for *P. distasonis*).

Estimating gene copy numbers on assembled genomes

Gene copies on a genome can severely influence SNV calling, as two similar copies of a gene may accumulate mutations over time. These fixed mutations differing between the two genes will be called as high confidence SNVs at 50% abundance, thus appearing as majority allele based on a random process in underlying reads covering the duplicated genes. For this we developed our own R scripts to use the coverage estimation across the species contigs in a 50 bp window (described above). Coverage across a contig is often not linear, due to growth of bacteria and a coverage gradient between the origin and terminus of replications [54].

Therefore, we first used a robust linear model (function rlm in R) to find a linear fit between coverage and genomic position. We corrected the genome coverage, using the residual of this linear model to obtain genome coverage estimates unobstructed by growth rate coverage dynamics. Within this fit, we identified positions using the arbitrary threshold > 1.7 (corrected)

average genome coverage for more than 4 consecutive 50 bp windows, to ensure that spurious variation in genome coverage was not flagged as SNVs.

Phylogenies estimated from consensus genomes per sample

The consensus genome per sample (as described above) was first further masked, at the beginning and end of contigs (200bp) and regions that were estimated to be copy number variations (see above). Samples were excluded if the amount of undefined genomic positions exceeded 60% of the total genome length. Further, for the non-synonymous trees, all sites except those at tRNA, rRNA or 0-fold sites within genes were removed from the alignment. For the synonymous trees only sites at 4-fold degenerate codon sites were included and the others removed from the alignment. From these consensus genomes a phylogenetic tree was built using the "buildTree4.pl" script from the MATAFILER pipeline. Briefly, for all sites we used gubbins [55] to remove recombining regions and reconstruct a phylogeny as implemented in the pipeline with the following command line options: "--filter_percentage 50 --tree_builder hybrid". For the non- and synonymous trees, we used IQ-TREE ver. 1.6.3.a [36] and ModelFinder [37] (option "-m TEST") to automatically determine the optimal nucleotide substitution model. To further validate our mutation rate estimates, we also constructed bootstrapped trees for non- and synonymous trees, using the option "--fast" to obtain 100 phylogenetic trees that were subsequently used to determine confidence intervals.

Statistical analysis

All statistical analysis was conducted in R 3.3.4 unless otherwise noted. Composition matrices such as species of ARG abundance matrices were normalized by sum, unless otherwise noted. Ordinations were visualized with non-metric multidimensional scaling (NMDS), as implemented in the vegan function "metaMDS", engine "monoMDS", using between sample Bray-Curtis distances, and restricted to two dimensions, unless otherwise noted. Ordination via principal coordinate analysis (PCoA) was calculated via the capscale function, as implemented in vegan. Differences between univariate variables such as taxonomic abundance or taxa richness were tested using a non-parametric Wilcoxon rank-sum test, with Benjamini-Hochberg multiple testing correction. Post-hoc statistical testing for significant differences between all combinations of three or more groups was conducted only for taxa with $p<0.2$ in the Kruskal-Wallis test. For this, wilcoxon rank-sum tests were calculated for all possible group combinations and corrected for multiple testing using Benjamini-Hochberg multiple testing correction.

Species richness was calculated using rtk [30], rarefying the species abundance matrix to 1200 species counts per sample, unless otherwise noted. Species nestedness and turnover was calculated using betapart 3.4.4 [56], from the species abundance matrix rarefied to 1200 counts per sample.

Species association network

The species association network was built from the normalized species abundance matrix, excluding species occurring in less than two samples. From this abundance matrix, a cooccurrence network was constructed using the meinshausen-buhlmann's neighborhood selection implemented in the R pipeline Spiec-Easi 0.1.4 [57], that relies on sparse neighborhoods and inverse covariance selection to construct its association network, to avoid detection of indirect associations. From the inferred network, we used igraph library version 1.2.1 [58] to remove loops and visualize the network. As edge weights, we used spearman correlations between single species.

Data availability. All metagenomics and metabarcoding sequences have been deposited in the European Bioinformatics Institute-Sequence Read Archive database, under accession number PRJEB28730.

Code availability. The C++ program to rarefy matrices is available under https://github.com/hildebra/Rarefaction. The pipeline to process metabarcoding sampels is available under http://psbweb05.psb.ugent.be/lotus/. The pipeline to process shotgun metagenomic samples is available under https://github.com/hildebra/MATAFILER .The C++ program to calculate read depth windows is available under https://github.com/hildebra/rdCover.

Supplemental Text

Genome binning assisted by different sequencing technologies and algorithms

^{U}*B. ceftriaxensis* appears to be a common rare member of the human gut microbiota, also found in other human associated environments. Since all culturing attempts failed (see section Methods), we had to reconstruct its genome from metagenomes in order improve our understanding of the species' biology. A subset of metagenomic samples that had a relative abundance of ^{U}*B. ceftriaxensis* >= 0.2% were selected and used in all subsequent metagenomic analysis (132 samples total, Suppl. Table 8), although even in this subset ^{U}*B. ceftriaxensis* was

often just above the metagenomic detection threshold (Suppl. Fig. 1b), demonstrating its rareness. These 132 metagenomes were *de novo* assembled and a novel gene catalog was constructed at a 95% gene identity threshold, totaling 5,790,967 non-redundant genes (see Methods). Through an initial draft genome binning of sample HD.S1.374, four species bins were recovered (Fig. 1a), consisting of ^{U}*B. ceftriaxensis*, as well as *Lactobacillus casei*, *Streptococcus thermophilus* and *Propionibacterium freudenreichii*. The GC vs abundance plots for days 347, 376, 377, 378 and 380 illustrating these species are show in Suppl. Fig. 17. The assembly quality of the three latter species was low, and those bins were therefore not used in further analysis (data not shown). Further, we recovered an initial binning of *P. distasonis* for sample HD.S1.377. Since the genomes were instable when rarefying to different sequencing depths, we used additionally canopy clustering and identified the bins representing these five species (Nielsen et al. 2014). Since predicted bins often differed by hundreds of genes (Suppl. Fig. 16c), a custom binning method starting from these two bins (Suppl. Fig. 16a,b, see Methods) was combined with a mate-pair sequencing library generated from sample HD.S1.380 (1.3% relative ^{U}*B. ceftriaxensis* abundance). Based on this, a draft genome for both ^{U}*B. ceftriaxensis* and *P. distasonis* was obtained that were used for further analysis unless otherwise mentioned. The ^{U}*B. ceftriaxensis* genome consisted of 8 scaffolds (10 contigs) with a N50 of 1,925,355 bp and a total length of 2,608,799 bp (see Suppl. Table 3). The genome contains 2,386 predicted genes, one SSU rRNA, two LSU rRNA and 45 tRNA genes as well as all 40 essential and conserved marker genes [14]. The 16S rRNA gene predicted in these contigs classified the species as part of the Firmicutes phylum, as detailed later. Within the set of NCBI unclassified environmental nucleotide sequences, we found a >99% identical sequence 16S rRNA gene sequence published in 2006, sequenced from fecal human samples [59]. This independently derived 16S rRNA gene sequence from stool samples gives an initial indication that the species does exist in other samples and is widely spread.

The ^{U}*B. ceftriaxensis* genome assembly was 94.6% complete based on the MiGA algorithm [33] containing 105/111 essential marker genes, of which two were potential duplicates (1.8% contamination). These estimates were reproducible with the CheckM algorithm [32] (94.8% completeness, contamination rate of 2.45%). The three most closely related genomes (see also Suppl. Fig. 5) in public databases were MGS (metagenomic species) based on metagenomic binning approaches [17]. Their binning quality appeared to be worse with an average genome completeness of 92% and a contamination of 1.04% (CheckM, Suppl. Table 4), which is still a fairly high quality for metagenomic assembled genomes [60]. Overall, the genome quality of the

new species can be considered as “high-quality-draft”, based on criteria put forward in [61], by far outranking the quality criteria proposed for draft genomes used to describe novel species (80% completeness, < 5% contamination)[60]. To investigate this further, a reference database of Firmicutes genomes was built, containing one randomly selected genome from most known Firmicutes species (total of 243 high quality genomes) referred to in the following as Firmicutes database. The genome quality of ^{U}*B. ceftriaxensis* is within the range of these reference genomes (genome completeness: 98.2± 4%, contamination 1.3±6.4%, mean ± sd for cultured Firmicutes species, Suppl. Table 4). The assembled genome of *P. distasonis* is of even higher quality (completeness 99.42%, contamination 0.38%, based on checkM).

Functional annotation indicates an anaerobic spore forming fermenter of a wide array of carbohydrates

Metabolism & Genetics

Functional annotation indicates that ^{U}*B. ceftriaxensi* is anaerobe with fermentative metabolism and able to utilize a wide range of sugars (detailed listing in Suppl. Table 6, see also Suppl. Fig. 2 for an overview). The carbohydrate-active enzyme (CAZyme) profile of ^{U}*B. ceftriaxensi* is dominated by enzymes acting on plant carbohydrates (60 genes), followed by animal derived/prevalent carbohydrates (24 genes). The low ratio of detectable signal peptides in CAZymes (28 of 269) indicates the limmited capacity of ^{U}*B. ceftriaxensi* processing dietary polysaccharides, which requires export of carbohydrate-active enzymes to the external milieu [58]. The CAZymes translocated by ^{U}*B. ceftriaxensi* that are related to dietary polysaccharides include glucosidades (GH13, GH3), fructofuranosidases (GH32) and galactosidades (GH36). ^{U}*B. ceftriaxensi* encodes components of ABC transporters for mono and oligosaccharides (103 genes). Their encoding proteins were annotated to participate in the uptake of multiple sugars, raffinose, stachyose, melibiose, N-acetylglucosamine, arabinogalactan oligomer and ribose. The preference for mono- and dissaccarides is supported by the presence of enzymes for the metabolism of alpha-L-fucoside, alpha-L-rhamnoside, alpha-trehalose, beta-glucoside, cellobiose, fructan, galactoside, L-arabinose, maltose, mannosides and mannose, sucrose, and xylan. Many of these sugars are directed to the glycolysis pathway (Embden-Meyerhof-Parnas), with exception of xylose and L-arabinose, which are directed to the Pentose Phosphate pathway (PPP). Genes of the oxidative part of pentose phosphate pathway (conversion of β-D-glucose 6-phosphate to D-ribulose 5-phosphate) were not detected. On the other hand, the non-oxidative part is almost complete, lacking only the transaldolase (EC: 2.2.1.2). This is indicative that fructose 6-phosphate and Glyceraldehyde 3-phosphate, but not glucose 6-phosphate, are the

connecting metabolites between glycolysis and PPP. As a central point in fermentative metabolism, pyruvate can be converted to ethanol and lactate. Pyruvate can also be converted to acetyl-coenzyme-A (acetyl-CoACoA), which is branching point for lipid synthesis and synthesis of TCA components, available for further anaplerotic reactions. This conversion is carried out by formate C-acetyltransferase, of which reversible reaction requires CoA and produces additionally formate. Considering the absence of genes encoding for formate hydrogenlyase, that disproportionates formate to CO2 and H2 [62], formate is likely incorporated into the one carbon pool by formate-tetrahydrofolate ligase or secreted [63]. Acetate can be converted to acetyl-CoA, via acetyl-CoA synthetase. Acetate consumption and production can be switched depending on the rate of acetate-producing substrates and acetates in the environment [64]. This could indicate a mixed acid fermentation.

It has been proposed a net of cross-feeding interactions for anaerobic conversion of dietary insoluble carbohydrates [65]. In this context, ^{U}*B. ceftriaxensi* would benefit from the extracellular degradation of polysaccharides by primary degraders and polysaccharide utilizers. In return, the products of fermentation of ^{U}*B. ceftriaxensi* would fuel the metabolism of other microbiome members, such as those that ferment lactate to butyrate [66]. In support of this scenario, 250 of the genes were involved in transport functions, including 182 genes coding for components of ABC transporters. In addition to genes encoding for carbohydrate transporters (described in the previous paragraph), ^{U}*B. ceftriaxensi* encodes transporter genes involved in the transport of vitamins, e.g. vitamin B12, minerals, drugs, phosphate and other compounds. Besides drug efflux transporters, Tec2 encodes 10 additional genes involved in drug resistance, including genes with putative beta-lactamase domains.

^{U}*B. ceftriaxensis* encodes two ATPases, a F-type and a V/A-type. Because of the absence of genes for oxidative phosphorylation proteins, it is likely that the F-type ATPase hydrolyses ATP to transport protons or sodium [67]. Therefore, both F-type and V/A-type, as V/A-type ATPase which is a proton or sodium pump, may function in pH homeostasis of the cell [68].

Interestingly, the ^{U}*B. ceftriaxensi* genome encodes proteins similar to structural components of microcompartments. Bacterial microcompartments are protein-bound organelles that confine cytotoxic intermediates [69] or enzymatic reactions compromised by oxygen [70]. As known for *Salmonella typhimurium* [71], it is likely that ^{U}*B. ceftriaxensi* microcompartments are involved in the utilization of 1,2 propanediol, which is a product of the fermentation of the sugars rhamnose and fucose, both naturally occurring in plants and other organism. Although the key enzyme of 1,2 propanediol utilization was not annotated in the genome, i.e. adenosylcobalamin-dependent diol dehydratase [72], the colocalization and co-orientation of microcompartments genes with

genes encoding rhamnose and fucose degrading enzymes supports the use of 1,2 propanediol in ^{U}*B. ceftriaxensi*. Specifically, co-localized genes include L-fuculose-phosphate aldolase and rhamnulokinase which are part of enzymatic reactions chains that produce (S)-lactaldehyde, that is reduced to 1,2 propanediol. This genomic arrangement is similar to what is found in other bacteria, where bacterial microcompartment (BMC) genes are colocalized with genes encoding for auxiliary functions, including, transporters and auxiliary genes [66]. Propanol and propionate are products of 1,2-propanediol utilization in bacteria [71,73].

^{U}*B. ceftriaxensi* is very likely a spore former, as it encodes 40 genes related to several stages of sporulation, including genes related to the stages II, III, IV and V. The species seems to be competent, i.e. recombination competent, as part of machinery required for competence (PilB, PilC, ComEC and ComFC) is present in the genome [74]. Beyond this competence, we find several genes associated to the defense against foreign DNA, such as restriction type I and 5-methylcytosine-specific restriction enzyme B.

Resistances and Pathogenicity

Although the metabolism of ^{U}*B. ceftriaxensi* seems to function anaerobic, it's possible that it can tolerate some degree of oxygenation, as we can find defense genes against reactive oxygen species (ROS), like rubrerythrin and thioredoxin. However, this could also be related to defense against white blood cells, that can use ROS to control bacteria [75].

We could assign 359 genes to either the Virulence Factor database (VFDB) [76] or a custom virulence factor subset from PATRIC [24]. However, most of these assignments do refer to parts of the bacterial metabolism. Several endotoxins seem to be present (lipopolysaccharide and lipooligosaccharides), genes involved in biofilm formation, O_2 resistance and genes involved in antiphagocytosis. Several genes are putatively involved in iron, copper and zinc uptake. Of note is further Haemolysin and corresponding transporters, required to lyse eukaryotic cells.

Comparing to the expected distribution given other Firmicutes bacteria, we observed a n.s. enrichment in the putative virulence categories Autophagy, Phosphate uptake, Escape from phagosome and Chaperone (data not shown).

Resistance of ^{U}*B. ceftriaxensis* to beta-lactam antibiotics

^{U}*B. ceftriaxensis* encodes genes related to drug resistance, such as genes coding for mate efflux family proteins, putative beta-lactamases and phosphinothricin acetyltransferase, which confer resistance to herbicides in *Streptomyces hygroscopicus* [77].

We further tested the two putative beta-lactamases experimentally, that had a low similarity to known beta-lactamases (33% and 31% AA identity, respectively to NCBI NR hypothetical beta-lactamases). Both candidate genes seem to be part of the core genome (peg.343, peg.1661, Suppl. Table 6), as they were assembled into the largest contig.

To determine the beta-lactamase activity of these genes, they were expressed in *E. coli* and beta-lactamase activity was tested on nitrocefin disks (Sigma). The disks changed color when they had contact with the CTX-M 15 beta-lactamase expressing colony used as positive control indicating strong beta-lactamase activity. A weaker, but clearly positive reaction was observed for ^{U}*B. ceftriaxensis* fig|6666666.214148.peg.343 (Suppl. Table 6). *E. coli* expressing ^{U}*B. ceftriaxensis* fig|6666666.214148.peg.2120 revealed no such activity (data not shown).

However, while the CTX-M 15 beta-lactamase led to ampicillin and ceftriaxone resistance in *E. coli,* none of the two potential ^{U}*B. ceftriaxensis* beta-lactamases was functional enough to support growth in presence of ampicillin or ceftriaxone.

Survival of ^{U}*B. ceftriaxensis* upon antibiotics treatment

Ceftriaxone disrupts the peptidoglycan synthesis in the bacterial cell wall, inhibiting essential penicillin binding proteins (pbp's). Bacterial resistance to beta-lactam antibiotics can be conferred either through resistant forms of pbp's, beta lactamases or through spore formation that survive the antibiotic treatment. ^{U}*B. ceftriaxensis* had two putative beta lactamases, but both showed no or insufficient activity against ceftriaxone experimentally (see above).

Alternatively, ^{U}*B. ceftriaxensis* might survive as dormant persistor cells or spores, such as *C. difficile* [78], as supported by high abundance of genes related to spore formation.

However, spore formation, that is persistence without growth, also seems unlikely as sole explanation, since ^{U}*B. ceftriaxensis* absolute cell numbers were >1,100x increased compared to baseline levels on the first day after antibiotic treatment. One would still expect a remaining ceftriaxone concentration of 12-25% in the gut at this time point (see Methods). Thus, a direct cephalosporine resistance of ^{U}*B. ceftriaxensis* seems likely, and this resistance is in gram positive bacteria often conferred through resistant pbp' that are inherently resistance to beta-lactams [79]. The ^{U}*B. ceftriaxensis* genome encodes two class 5/6 pbp's, a class that has been linked to confer cephalopsporin resistance in *Enterococci* and *Listeria* [80,81]. Before, it has been shown to confer specifically ceftriaxone resistance [82]. While there are no molecular tools to test the resistance of this pbp by transfer of the gene into another species, it seems most plausible that ^{U}*B. ceftriaxensis* is resistant to ceftriaxone and that this resistance might be intrinsically conferred through a combination of resistant pbp's and surviving as spores.

Detection of ^{U}*B. ceftriaxensis* in public samples

To investigate the prevalence of ^{U}*B. ceftriaxensis* in diverse environments we screened 3,692 public metagenomic datasets from human associated and other environments (see Methods). The overall prevalence of ^{U}*B. ceftriaxensis* was 20.1% over all samples (Suppl. Table 7), being most prevalent in gut samples from 3 continents (30.1% of 1,801) but entirely absent in ocean samples (n=243). Exploring different human body sites, it is detectable in 8 out of 18 non-gut sites. However, in non-gut samples detection rate was spurious (2.6% of 952 non-gut human associated samples). We further analyzed 1,163 metagenomes balanced among animal guts (mouse, pig, cat and dog), as well as ocean and soil, but did not detect this species in any of these.

Density of bacterial cells in stool samples

Cell numbers are relatively stable within and between patients, ranging from 0.11 - 1.03 e12 cells/ml stool. This seems realistic, although slightly higher than the average reported in a metanalysis of several studies (0.92e11 cells/ml stool, [83]). Since our cell counts were restricted to 19 samples total, we had no overlapping time point between pre-antibiotic ^{U}*B. ceftriaxensis* detection and cell counts. To still infer absolute ratio change in ^{U}*B. ceftriaxensis* bloom at day 374 compared to previous samples, we used the relative abundance of the deeply sequenced day 7 and the mean abundance of all samples with cell counts before day 374, namely day 0 and 60 to approximate the absolute abundance of ^{U}*B. ceftriaxensis* before day 374.

***P. distasonis* patient specific association**

P. distasonis was associated to an antibiotic induced monodominance and therefore we wanted to investigate if *P. distasonis* was replaced by another strain of the same species during the antibiotic treatment. This analysis showed that *P. distasonis* remained stable over time in subject HD.S1, but also most other subjects in the discovery dataset, with the exception of two subjects that showed a clear change of *P. distasonis* genotypes (Suppl. Fig. 10a). Furthermore, we found that *P. distasonis* strains were nearly identical strains (<100 nt differences genome

wide) in subjects (HD.S1 + HD.S4, HD.S8 + HD.S9). We note that HD.S1+HD.S4, as well as HD.S8+HD.S9 are each family members and a within family strain transfer seems to be the most likely scenario explaining this similarity (Suppl. Fig. 10a).
Between patients, we found an average inter-sample evolutionary distance of 99.1±0.5% (corresponding to 45,840 nt average genomewide differences, Suppl. Fig. 10b). This indicates that this species could be represented by a single ecotype within the 31 subjects analyzed here.

Functional changes upon antibiotics treatment

We hypothesized that functional potential of the microbiome should be changed after the antibiotic treatment. Indeed, putative pathogenicity genes (PPGs) were increased in the first and decreased in the second stage, relative to states after >30 days of treatment, while for antibiotic resistance genes (ARG) the opposite was true (Fig. 4c, $p<0.05$, Suppl. Table 12): the second stage was significantly enriched in ARGs, specifically beta-lactamases (Suppl. Table 13), that confer a resistance to both antibiotics used. PPGs were enriched in the first stage. In the second stage overall CAZy potential was increased ($p=0.0028$), while it was decreased in the first stage (Fig. 4c). CAZymes categories specific to food derived (plants, animals) carbohydrates were increased during both second stages, especially after Cefuroxime treatment (Suppl. Fig. 13a), in contrast to decreased CAZYmes specific to microbial substrates (bacterial, fungal) (Suppl. Fig. 13b). This could be interpreted as predatory species being decreased in the first stage of community rebuilding.

Monodominance occurrence in the human microbiome

While monodominance can be considered an unnatural state of the healthy human microbiome by our current knowledge, the occurrence of monodominance could be higher than currently published studies would imply. Conversely we argue, that monodominance states might have been overlooked so far, as (i) temporal sampling is usually much sparser than our study; (ii) 16S sequencing might underestimate the true abundance of monodominant species due to PCR biases and 16S copy number variations [84,85]; (iii) only few microbial studies have included patients treated with intravenous antibiotics [86] [87]; (iv) samples might have been discarded from analysis due to “contamination” with a single unknown organism, which standard metagenomics methods will often fail to detect [40,88] and (v) such dramatic community restructuring can remain unnoticed as subject HD.S1 did not report any phenotypic effects during monodominant phases.

Proposed changes to firmicutes taxonomy and placement of [U]*B. ceftriaxensis*- supporting information

In the high resolution phylogeny for Firmicutes taxa, most families appear monophyletic, but a few notable deviations can be observed in the phylogeny based on 40 marker genes (Fig. 3) or in the 16S based phylogeny (Suppl. Fig. 6). For example, *Eubacterium rectale* is clearly in the same clade as Lachnospiraceae and not Eubacteriaceae as proposed in the NCBI taxonomy and therefore recently proposed to be reclassified to family Lachnospiraceae, based on genomic as well as cellular features [89], as also in our phylogeny. *Heliobacterium modesticaldum* seems to either be part of Peptococcaceae, several species currently classified as Peptococcaceae are a new family, based on both 40 MG and 16S phylogeny. We have proposed several suggested changes to the Firmicutes taxonomy in Suppl. Table 10. Indeed, a recent work describing most bacterial genomes available publicly proposed very similar changes [90]. Of note are Thermoacaerobacteraceae and Peptococcaceae, that appear intermixed based on the 40 MGs phylogeny (Fig. 3), though this is only to a lesser degree supported by the 16S rRNA gene phylogeny.

The family Ruminococcaceae is especially interesting in our analysis, as [U]*B. ceftriaxensis* could be part of this clade. Notably, Ruminococcaceae is paraphyletic based on our analysis, and we propose to split this family into three families: there is a distinct “core” group including genera such as *Faecalibacterium* and the type genus *Ruminococcus*, that are monophyletic in all phylogenetic analysis. However, other Ruminococcaceae can be separated into two clusters: *Clostridium cellulyticum*, *Clostridium josui*, *Pseudobacteroides cellulosolvens*, *Ruminiclostridium thermocellum* and *Acidothermus cellulyticus* form an outgroup that is well supported by bootstrap values and always separated from the core Ruminococcaceae clade, by either the family Eubacteriaceae (Fig. 3) or the family Oscillospiraceae (Suppl. Fig. 6). Intriguingly, *R. thermocellum* was recently renamed from Clostridium to genus Ruminiclostridium, with the authors already noting its uncertain phylogenetic position in family Ruminococcaceae [91]. The third Ruminococcaceae clade consists of *Mageeibacillus indolicus*, recently isolated from human vaginal samples [92], *Ruminococcaceae bacterium* AB4001 and *Ruminococcaceae bacterium* AE2021, both part of the Hungate 1000 project [2] culturing cow rumen bacteria. To resolve the observed polyphyletic branching of Ruminococcaceae, we propose that the two discussed clades should form two new taxonomic families by themselves (Suppl. Table 10),

[2] http://www.rmgnetwork.org/hungate1000.html

named **Erisaceae** fam. nov. and **Discordiaceae**., fam. nov., named after the greek and latin goddess of discord, Eris and Discordia. It is of note that AB4001 and AE2021 fall only within the Discordiaceae family in the 40 MG tree, while their 16S seems to place them within Lachnospiraceae (Suppl. Fig. 6).
This grouping is further supported by distance-based analysis: In our phylogenetic analysis we argue that the polyphyletic Ruminococcaceae family represents multiple clades, based on phylogenetic maximum likelihood trees (16S, marker genes). We propose to divide Ruminococcaceae into different families with ^{U}*B. Ceftriaxensis* placed as a new separate family.

The proposed phylogenetic structure was further tested based on the similarity (percent identity) of the 40 marker genes nucleotide sequences, amino acid sequences and 16S sequences. As a reference, the minimum and the median percent identities from genomes of our Firmicutes reference database were calculated on different taxonomic levels, restricted to species that represent monophyletic clades in the amino acid tree. We compared the genomic similarity of Ruminococcaceae species to each other and to within family genetic similarities of other Firmicutes families (Suppl. Table. 9). First, we note that the median amino acid as well as 16S nucleotide similarity of all members of the originally annotated Ruminococcaceae family is very low (60.7% and 88.8%) compared to the average among all other Firmicutes families (70.9% and 91.6%), actually even lower or comparable to the average within class level (62.1% and 88.2%) strongly indicating that species currently annotated as Ruminococcaceae should be represented by multiple families, or even classes. This agrees with our phylogenetic analysis (see above), where we identified three distinct clusters of Ruminococcaceae: the core cluster composed of seven species and *Ruminococcus sp.*, the three species of Discordiaceae fam. nov. and Eriseae fam. nov.. While the Eriseae family clearly represents one family (71.7% median amino acid identity), the members of the Ruminococcaceae main cluster and the Discordieae cluster are both less similar (65.4% and 63.3% median amino acid identity, respectively), thus potentially still representing more than one family, given 70.9% being on average the median AA similarity typically found among members of Firmicutes families (Suppl. Table 9, Suppl. Fig. 5).

Further, our analysis strongly indicates that ^{U}*B. ceftriaxensis* represents a new separate family including three MGS, binned genomes from gut metagenomic samples (Nielsen et al. 2014). This clade is in our analysis distinct from Ruminococcaceae, Discordieae and Eriseae, given median genetic similarities (Suppl. Fig. 5) and phylogeny (Fig. 3). Instead family

Catabacteriaceae is the most similar family, represented by type species *Catabacter hongkongensis*.

Bibliography (Methods & Supplement)

1 Voigt AY, Costea PI, Kultima JR, *et al.* Temporal and technical variability of human gut metagenomes. *Genome Biol* 2015;**16**:73. doi:10.1186/s13059-015-0639-8

2 Plugge CM. Anoxic media design, preparation, and considerations. *Methods Enzymol* 2005;**397**:3–16. doi:10.1016/S0076-6879(05)97001-8

3 Yilmaz P, Parfrey LW, Yarza P, *et al.* The SILVA and 'All-species Living Tree Project (LTP)' taxonomic frameworks. *Nucleic Acids Res* 2014;**42**:D643-8. doi:10.1093/nar/gkt1209

4 Schramm A, Fuchs BM, Nielsen JL, *et al.* Fluorescence in situ hybridization of 16S rRNA gene clones (Clone-FISH) for probe validation and screening of clone libraries. *Environ Microbiol* 2002;**4**:713–20.http://www.ncbi.nlm.nih.gov/pubmed/12460279

5 Puente-Sanchez F, Aguirre J, Parro V, *et al.* A novel conceptual approach to read-filtering in high-throughput amplicon sequencing studies. *Nucleic Acids Res* 2015;:gkv1113-. doi:10.1093/nar/gkv1113

6 Hildebrand F, Tadeo R, Voigt A, *et al.* LotuS: an efficient and user-friendly OTU processing pipeline. *Microbiome* 2014;**2**:30. doi:10.1186/2049-2618-2-30

7 Wood DE, Salzberg SL. Kraken: ultrafast metagenomic sequence classification using exact alignments. *Genome Biol* 2014;**15**:R46. doi:10.1186/gb-2014-15-3-r46

8 Rasmussen S, Allentoft ME, Nielsen K, *et al.* Early Divergent Strains of Yersinia pestis in Eurasia 5,000 Years Ago. *Cell* 2015;**163**:571–82. doi:10.1016/j.cell.2015.10.009

9 Li H. Toward better understanding of artifacts in variant calling from high-coverage samples. *Bioinformatics* 2014;**30**:2843–51. doi:10.1093/bioinformatics/btu356

10 Bankevich A, Nurk S, Antipov D, *et al.* SPAdes: a new genome assembly algorithm and its applications to single-cell sequencing. *J Comput Biol* 2012;**19**:455–77. doi:10.1089/cmb.2012.0021

11 Hyatt D, Chen G-L, Locascio PF, *et al.* Prodigal: prokaryotic gene recognition and translation initiation site identification. *BMC Bioinformatics* 2010;**11**:119. doi:10.1186/1471-2105-11-119

12 Fu L, Niu B, Zhu Z, *et al.* CD-HIT: Accelerated for clustering the next-generation sequencing data. *Bioinformatics* 2012;**28**:3150–2. doi:10.1093/bioinformatics/bts565

13 Langmead B, Salzberg SL. Fast gapped-read alignment with Bowtie 2. *Nat Methods* 2012;**9**:357–9. doi:10.1038/nmeth.1923

14 Mende DR, Sunagawa S, Zeller G, *et al.* Accurate and universal delineation of prokaryotic species. *Nat Methods* 2013;**10**:881–4. doi:10.1038/nmeth.2575

15 Hauswedell H, Singer J, Reinert K. Lambda: the local aligner for massive biological data. *Bioinformatics* 2014;**30**:i349–55. doi:10.1093/bioinformatics/btu439

16 Mende DR, Letunic I, Huerta-Cepas J, *et al.* proGenomes: a resource for consistent functional and taxonomic annotations of prokaryotic genomes. *Nucleic Acids Res* 2016;**45**:gkw989. doi:10.1093/nar/gkw989

17 Nielsen HB, Almeida M, Juncker AS, *et al.* Identification and assembly of genomes and genetic elements in complex metagenomic samples without using reference genomes. *Nat Biotechnol* 2014;**32**:822–8. doi:10.1038/nbt.2939

18 Li H, Handsaker B, Wysoker A, *et al.* The Sequence Alignment/Map format and

SAMtools. *Bioinformatics* 2009;**25**:2078–9. doi:10.1093/bioinformatics/btp352
19 Buchfink B, Xie C, Huson DH. Fast and sensitive protein alignment using DIAMOND. *Nat Methods* Published Online First: 17 November 2014. doi:10.1038/nmeth.3176
20 Cantarel BL, Coutinho PM, Rancurel C, *et al.* The Carbohydrate-Active EnZymes database (CAZy): an expert resource for Glycogenomics. *Nucleic Acids Res* 2009;**37**:D233–8. doi:10.1093/nar/gkn663
21 Kanehisa M, Goto S. KEGG: kyoto encyclopedia of genes and genomes. *Nucleic Acids Res* 2000;**28**:27–30.
22 McArthur AG, Waglechner N, Nizam F, *et al.* The comprehensive antibiotic resistance database. *Antimicrob Agents Chemother* 2013;**57**:3348–57. doi:10.1128/AAC.00419-13
23 Huerta-Cepas J, Szklarczyk D, Forslund K, *et al.* eggNOG 4.5: a hierarchical orthology framework with improved functional annotations for eukaryotic, prokaryotic and viral sequences. *Nucleic Acids Res* 2016;**44**:D286-93. doi:10.1093/nar/gkv1248
24 Wattam AR, Davis JJ, Assaf R, *et al.* Improvements to PATRIC, the all-bacterial Bioinformatics Database and Analysis Resource Center. *Nucleic Acids Res* 2017;**45**:D535–42. doi:10.1093/nar/gkw1017
25 Cantarel BL, Lombard V, Henrissat B. Complex Carbohydrate Utilization by the Healthy Human Microbiome. *PLoS One* 2012;**7**:e28742. doi:10.1371/journal.pone.0028742
26 Cardenas E, Kranabetter JM, Hope G, *et al.* Forest harvesting reduces the soil metagenomic potential for biomass decomposition. *ISME J* 2015;**9**:2465–76. doi:10.1038/ismej.2015.57
27 Forslund K, Hildebrand F, Nielsen T, *et al.* Disentangling type 2 diabetes and metformin treatment signatures in the human gut microbiota. *Nature* 2015;**528**:262–6. doi:10.1038/nature15766
28 Vieira-Silva S, Rocha EPC. The systemic imprint of growth and its uses in ecological (meta)genomics. *PLoS Genet* 2010;**6**:e1000808. doi:10.1371/journal.pgen.1000808
29 Wu Y-W, Tang Y-H, Tringe SG, *et al.* MaxBin: an automated binning method to recover individual genomes from metagenomes using an expectation-maximization algorithm. *Microbiome* 2014;**2**:26. doi:10.1186/2049-2618-2-26
30 Saary P, Forslund K, Bork P, *et al.* RTK: efficient rarefaction analysis of large datasets. *Bioinformatics* 2017;:1–2. doi:10.1093/bioinformatics/btx206
31 Sahlin K, Vezzi F, Nystedt B, *et al.* BESST - Efficient scaffolding of large fragmented assemblies. *BMC Bioinformatics* 2014;**15**:281. doi:10.1186/1471-2105-15-281
32 Parks DH, Imelfort M, Skennerton CT, *et al.* CheckM: assessing the quality of microbial genomes recovered from. *Cold Spring Harb Lab Press Method* 2015;**1**:1–31. doi:10.1101/gr.186072.114
33 Rodriguez-R LM, Gunturu S, Harvey WT, *et al.* The Microbial Genomes Atlas (MiGA) webserver: taxonomic and gene diversity analysis of Archaea and Bacteria at the whole genome level. *Nucleic Acids Res* 2018;**46**:W282–8. doi:10.1093/nar/gky467
34 Lagesen K, Hallin P, Rødland EA, *et al.* RNAmmer: consistent and rapid annotation of ribosomal RNA genes. *Nucleic Acids Res* 2007;**35**:3100–8. doi:10.1093/nar/gkm160
35 Sievers F, Wilm A, Dineen D, *et al.* Fast, scalable generation of high-quality protein multiple sequence alignments using Clustal Omega. *Mol Syst Biol* 2011;**7**:539. doi:10.1038/msb.2011.75
36 Nguyen L-T, Schmidt HA, von Haeseler A, *et al.* IQ-TREE: A Fast and Effective Stochastic Algorithm for Estimating Maximum-Likelihood Phylogenies. *Mol Biol Evol* 2015;**32**:268–74. doi:10.1093/molbev/msu300
37 Kalyaanamoorthy S, Minh BQ, Wong TKF, *et al.* ModelFinder: fast model selection for accurate phylogenetic estimates. *Nat Methods* 2017;**14**:587–9. doi:10.1038/nmeth.4285
38 Lemoine F, Domelevo Entfellner J-B, Wilkinson E, *et al.* Renewing Felsenstein’s phylogenetic bootstrap in the era of big data. *Nature* 2018;**556**:452–6.

doi:10.1038/s41586-018-0043-0

39 Letunic I, Bork P. Interactive tree of life (iTOL) v3: an online tool for the display and annotation of phylogenetic and other trees. *Nucleic Acids Res* 2016;**44**:W242–5. doi:10.1093/nar/gkw290

40 Sunagawa S, Mende DR, Zeller G, *et al.* Metagenomic species profiling using universal phylogenetic marker genes. *Nat Methods* 2013;**10**:1196–9. doi:10.1038/nmeth.2693

41 Costea PI, Coelho LP, Sunagawa S, *et al.* Subspecies in the global human gut microbiome. *Mol Syst Biol* 2017;**13**:960. doi:10.15252/msb.20177589

42 Feng Q, Liang S, Jia H, *et al.* Gut microbiome development along the colorectal adenoma-carcinoma sequence. *Nat Commun* 2015;**6**. doi:10.1038/ncomms7528

43 Qin J, Li Y, Cai Z, *et al.* A metagenome-wide association study of gut microbiota in type 2 diabetes. *Nature* 2012;**490**:55–60. doi:10.1038/nature11450

44 Huttenhower C, Gevers D, Knight R, *et al.* Structure, function and diversity of the healthy human microbiome. Nature. 2012;**486**:207–14. doi:10.1038/nature11234

45 Zeller G, Tap J, Voigt AY, *et al.* Potential of fecal microbiota for early-stage detection of colorectal cancer. *Mol Syst Biol* 2014;**10**:766.http://www.pubmedcentral.nih.gov/articlerender.fcgi?artid=4299606&tool=pmcentrez&rendertype=abstract (accessed 30 Nov 2014).

46 Karlsson FH, Tremaroli V, Nookaew I, *et al.* Gut metagenome in European women with normal, impaired and diabetic glucose control. *Nature* 2013;**498**:99–103. doi:10.1038/nature12198

47 Coelho LP, Kultima JR, Costea PI, *et al.* Similarity of the dog and human gut microbiomes in gene content and response to diet. *Microbiome* 2018;**6**:72. doi:10.1186/s40168-018-0450-3

48 Deusch O, O'Flynn C, Colyer A, *et al.* A longitudinal study of the feline faecal microbiome identifies changes into early adulthood irrespective of sexual development. *PLoS One* 2015;**10**:1–21. doi:10.1371/journal.pone.0144881

49 Sunagawa S, Coelho LP, Chaffron S, *et al.* Ocean plankton. Structure and function of the global ocean microbiome. *Science* 2015;**348**:1261359. doi:10.1126/science.1261359

50 Bahram M, Hildebrand F, Forslund SK, *et al.* Structure and function of the global topsoil microbiome. *Nature* 2018;**560**:233–7. doi:10.1038/s41586-018-0386-6

51 Willmann M, El-Hadidi M, Huson DH, *et al.* Antibiotic Selection Pressure Determination through Sequence-Based Metagenomics. *Antimicrob Agents Chemother* 2015;**59**:7335–45. doi:10.1128/AAC.01504-15

52 Kent WJ. BLAT--the BLAST-like alignment tool. *Genome Res* 2002;**12**:656–64. doi:10.1101/gr.229202

53 Garrison E, Marth G. Haplotype-based variant detection from short-read sequencing. 2012;:9.http://arxiv.org/abs/1207.3907 (accessed 11 Jul 2014).

54 Korem T, Zeevi D, Suez J, *et al.* Growth dynamics of gut microbiota in health and disease inferred from single metagenomic samples. *Science (80-)* 2015;**349**:1101–6. doi:10.1126/science.aac4812

55 Croucher NJ, Page AJ, Connor TR, *et al.* Rapid phylogenetic analysis of large samples of recombinant bacterial whole genome sequences using Gubbins. *Nucleic Acids Res* 2015;**43**:e15–e15. doi:10.1093/nar/gku1196

56 Baselga A, Orme CDL. Betapart: An R package for the study of beta diversity. *Methods Ecol Evol* 2012;**3**:808–12. doi:10.1111/j.2041-210X.2012.00224.x

57 Kurtz ZD, Müller CL, Miraldi ER, *et al.* Sparse and Compositionally Robust Inference of Microbial Ecological Networks. *PLOS Comput Biol* 2015;**11**:e1004226. doi:10.1371/journal.pcbi.1004226

58 Csardi G, Nepusz T. The igraph software package for complex network research. *InterJournal, Complex Syst* 2006;**1695**:1–9.

59 Ley RE, Turnbaugh PJ, Klein S, *et al.* Human gut microbes associated with obesity. *Nature* 2006;**444**:1022–3. doi:10.1038/nature4441022a
60 Konstantinidis KT, Rosselló-Móra R, Amann R. Uncultivated microbes in need of their own taxonomy. *ISME J* 2017;**11**:2399–406. doi:10.1038/ismej.2017.113
61 Bowers RM, Kyrpides NC, Stepanauskas R, *et al.* Minimum information about a single amplified genome (MISAG) and a metagenome-assembled genome (MIMAG) of bacteria and archaea. *Nat Biotechnol* 2017;**in press**:725–31. doi:10.1038/nbt.3893
62 Sawers G. The hydrogenases and formate dehydrogenases of Escherichia coli. *Antonie Van Leeuwenhoek* 1994;**66**:57–88.http://www.ncbi.nlm.nih.gov/pubmed/7747941
63 Leonhartsberger S, Korsa I, Böck A. The molecular biology of formate metabolism in enterobacteria. *J Mol Microbiol Biotechnol* 2002;**4**:269–76.http://www.ncbi.nlm.nih.gov/pubmed/11931558
64 Wolfe AJ. The acetate switch. *Microbiol Mol Biol Rev* 2005;**69**:12–50. doi:10.1128/MMBR.69.1.12-50.2005
65 Flint HJ, Duncan SH, Scott KP, *et al.* Interactions and competition within the microbial community of the human colon: links between diet and health. *Environ Microbiol* 2007;**9**:1101–11. doi:10.1111/j.1462-2920.2007.01281.x
66 Axen SD, Erbilgin O, Kerfeld CA. A Taxonomy of Bacterial Microcompartment Loci Constructed by a Novel Scoring Method. *PLoS Comput Biol* 2014;**10**:e1003898. doi:10.1371/journal.pcbi.1003898
67 Ferguson SA, Keis S, Cook GM. Biochemical and Molecular Characterization of a Na+-Translocating F1Fo-ATPase from the Thermoalkaliphilic Bacterium Clostridium paradoxum. *J Bacteriol* 2006;**188**:5045–54. doi:10.1128/JB.00128-06
68 Lolkema JS, Chaban Y, Boekema EJ. No Title. *J Bioenerg Biomembr* 2003;**35**:323–35. doi:10.1023/A:1025776831494
69 Sampson EM, Bobik TA. Microcompartments for B12-Dependent 1,2-Propanediol Degradation Provide Protection from DNA and Cellular Damage by a Reactive Metabolic Intermediate. *J Bacteriol* 2008;**190**:2966–71. doi:10.1128/JB.01925-07
70 Kerfeld CA, Aussignargues C, Zarzycki J, *et al.* Bacterial microcompartments. *Nat Rev Microbiol* 2018;**16**:277–90. doi:10.1038/nrmicro.2018.10
71 Obradors N, Badía J, Baldomà L, *et al.* Anaerobic metabolism of the L-rhamnose fermentation product 1,2-propanediol in Salmonella typhimurium. *J Bacteriol* 1988;**170**:2159–62.http://www.ncbi.nlm.nih.gov/pubmed/3283105
72 Bobik TA, Havemann GD, Busch RJ, *et al.* The propanediol utilization (pdu) operon of Salmonella enterica serovar Typhimurium LT2 includes genes necessary for formation of polyhedral organelles involved in coenzyme B(12)-dependent 1, 2-propanediol degradation. *J Bacteriol* 1999;**181**:5967–75.http://www.ncbi.nlm.nih.gov/pubmed/10498708
73 Petit E, LaTouf WG, Coppi M V., *et al.* Involvement of a Bacterial Microcompartment in the Metabolism of Fucose and Rhamnose by Clostridium phytofermentans. *PLoS One* 2013;**8**:e54337. doi:10.1371/journal.pone.0054337
74 Seitz P, Blokesch M. DNA-uptake machinery of naturally competent Vibrio cholerae. *Proc Natl Acad Sci* 2013;**110**:17987–92. doi:10.1073/pnas.1315647110
75 Sommer F, Moltzau Anderson J, Bharti R, *et al.* The resilience of the intestinal microbiota influences health and disease. *Nat Publ Gr* Published Online First: 2017. doi:10.1038/nrmicro.2017.58
76 Chen L, Zheng D, Liu B, *et al.* VFDB 2016: hierarchical and refined dataset for big data analysis—10 years on. *Nucleic Acids Res* 2016;**44**:D694–7. doi:10.1093/nar/gkv1239
77 Thompson CJ, Movva NR, Tizard R, *et al.* Characterization of the herbicide-resistance gene bar from Streptomyces hygroscopicus. *EMBO J* 1987;**6**:2519–23.http://www.ncbi.nlm.nih.gov/pubmed/16453790

78 Lawley TD, Clare S, Walker AW, *et al.* Antibiotic treatment of Clostridium difficile carrier mice triggers a supershedder state, spore-mediated transmission, and severe disease in immunocompromised hosts. *Infect Immun* 2009;**77**:3661–9. doi:10.1128/IAI.00558-09
79 Rice LB. Mechanisms of resistance and clinical relevance of resistance to β-lactams, glycopeptides, and fluoroquinolones. *Mayo Clin Proc* 2012;**87**:198–208. doi:10.1016/j.mayocp.2011.12.003
80 Krawczyk-Balska A, Markiewicz Z. The intrinsic cephalosporin resistome of Listeria monocytogenes in the context of stress response, gene regulation, pathogenesis and therapeutics. *J Appl Microbiol* 2016;**120**:251–65. doi:10.1111/jam.12989
81 Collins J, Robinson C, Danhof H, *et al.* Dietary trehalose enhances virulence of epidemic Clostridium difficile. *Nature* 2018;**553**:291–4. doi:10.1038/nature25178
82 Arbeloa A, Segal H, Hugonnet J-E, *et al.* Role of class A penicillin-binding proteins in PBP5-mediated beta-lactam resistance in Enterococcus faecalis. *J Bacteriol* 2004;**186**:1221–8. doi:10.1128/JB.186.5.1221
83 Sender R, Fuchs S, Milo R. Revised Estimates for the Number of Human and Bacteria Cells in the Body. *PLOS Biol* 2016;**14**:e1002533. doi:10.1371/journal.pbio.1002533
84 Bahram M, Anslan S, Hildebrand F, *et al.* Newly designed 16S rRNA metabarcoding primers amplify diverse and novel archaeal taxa from the environment. *Environ Microbiol Rep* 2018;**5**. doi:10.1111/1758-2229.12684
85 Tedersoo L, Anslan S, Bahram M, *et al.* Shotgun metagenomes and multiple primer pair-barcode combinations of amplicons reveal biases in metabarcoding analyses of fungi. *MycoKeys* 2015;**10**:1–43. doi:10.3897/mycokeys.10.4852
86 Surawicz CM. Antibiotic-associated diarrhea and pseudomembranous colitis: Are they less common with poorly absorbed antimicrobials? *Chemotherapy* 2005;**51**:81–9. doi:10.1159/000081993
87 Panda S, El khader I, Casellas F, *et al.* Short-Term Effect of Antibiotics on Human Gut Microbiota. *PLoS One* 2014;**9**:e95476. doi:10.1371/journal.pone.0095476
88 Truong DT, Franzosa EA, Tickle TL, *et al.* MetaPhlAn2 for enhanced metagenomic taxonomic profiling. *Nat Methods* 2015;**12**:902–3. doi:10.1038/nmeth.3589
89 Rosero JA, Killer J, Sechovcová H, *et al.* Reclassification of Eubacterium rectale (Hauduroy et al. 1937) prévot 1938 in a new genus agathobacter gen. nov. as Agathobacter rectalis comb. nov., and description of Agathobacter ruminis sp. nov., isolated from the rumen contents of sheep and cows. *Int J Syst Evol Microbiol* 2016;**66**:768–73. doi:10.1099/ijsem.0.000788
90 Parks DH, Chuvochina M, Waite DW, *et al.* A standardized bacterial taxonomy based on genome phylogeny substantially revises the tree of life. *Nat Biotechnol* 2018;:256800. doi:10.1038/nbt.4229
91 Yutin N, Galperin MY. A genomic update on clostridial phylogeny: Gram-negative spore formers and other misplaced clostridia. *Environ Microbiol* 2013;**6**:n/a-n/a. doi:10.1111/1462-2920.12173
92 Austin MN, Rabe LK, Srinivasan S, *et al.* Mageeibacillus indolicus gen. nov., sp. nov.: A novel bacterium isolated from the female genital tract. *Anaerobe* 2015;**32**:37–42. doi:10.1016/j.anaerobe.2014.12.003
