# Supplementary material for: Antibiotics-induced monodominance of a novel gut bacterial order
Source: Gut. 2019 Jan 18;68(10):1781–90. doi: 10.1136/gutjnl-2018-317715 (PMC6839795; doi:10.1136/gutjnl-2018-317715)
Supplement: Supplementary file 2 [file gutjnl-2018-317715supp002.pdf]

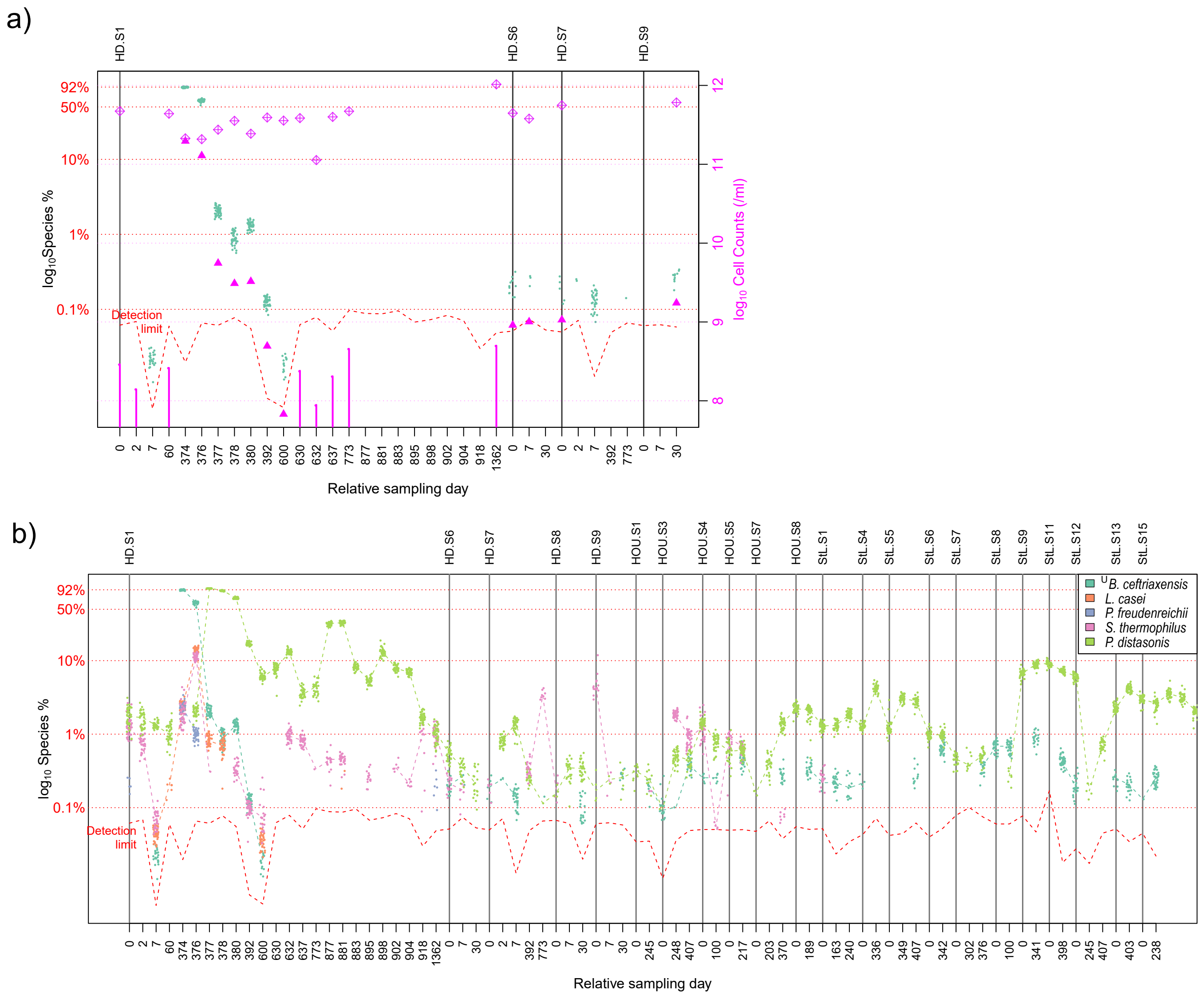

Suppl. Fig. 1: a) The relative abundance of ᵁ*B. ceftriaxensis* based on 40 conserved marker genes (green dots) is often close to the metagenomic detection limit (dashed red line). Pink dots are absolute total cell numbers measured via flow cytometry, pink solid lines show the possible abundance range, if the species could not be detected in that specific samples. Only individuals are shown for which in at least one time -point cell counts were measured (pink diamonds). From this we derive, that only bacteria with at least 2.3e8 cells per ml feces (median) can be detected via metagenomics. b) Time series of all samples with >0.2% ᵁ*B. ceftriaxensis* relative abundance, showing the conserved marker gene abundance of five select species.The average detection threshold was 0.061% (dashed line).

Suppl. Fig. 2: Overview of the core metabolism and transporters related to this for the new species, UB. ceftriaxensis. Note that this modelwas created based on genome assembly and bioinformatic annotation and extensive human curation (see Supplement).

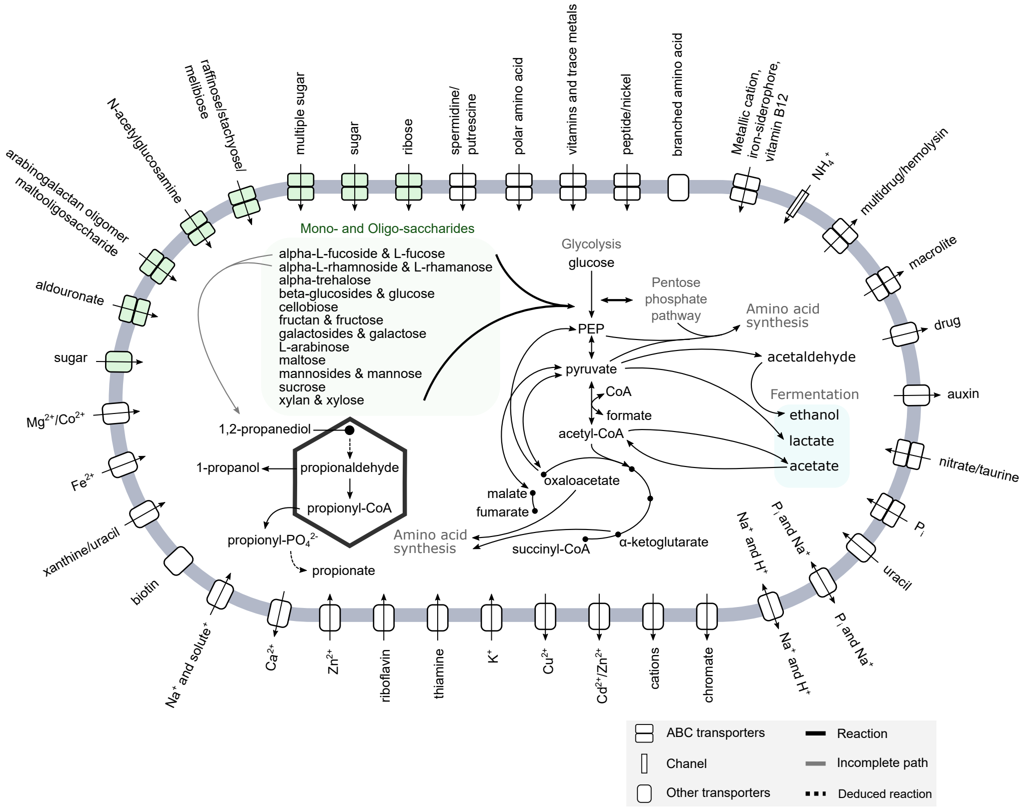

a)

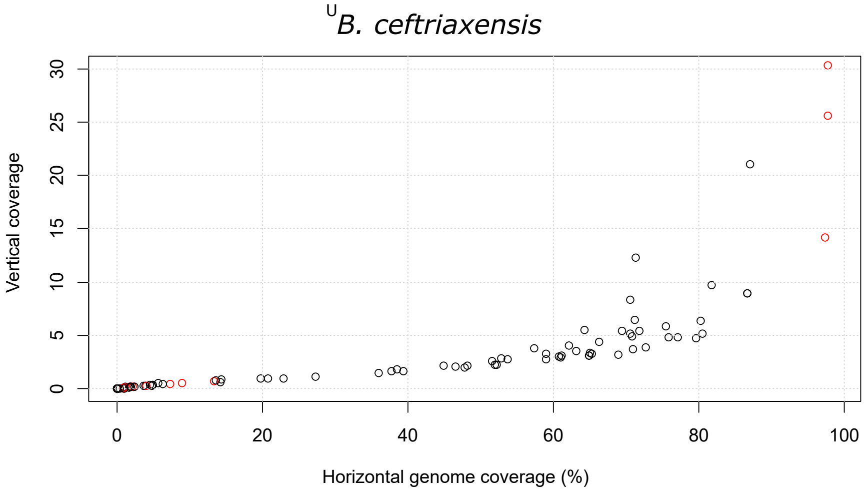

b)

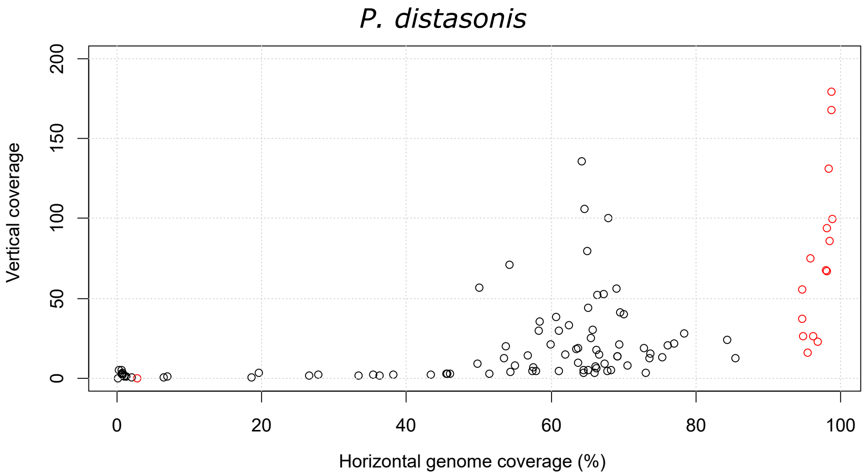

Suppl. Fig. 3: Comparison of verticalcoverage (average depth per base) to horizontal coverage (fraction of genome covered). This plot shows for a) ^{U}B. ceftriaxensis that the coverage is in general too low to infer patterns, while for b) *P. distasonis*the coverage was higher and it seems like a larger fraction of samples is only sharing ~60% of its genome with the *P. distasonis*strain assembled from HD.S1. Red points are samples from subject HD.S1, that were also used to assemble these two reference genomes, while black points represent samples from other subjectsin the reference set (see main text). Note that y-axis is limited and high coverage samples are not visible (these are all from HD.S1,all having > 95% genome coverage in both graphs).

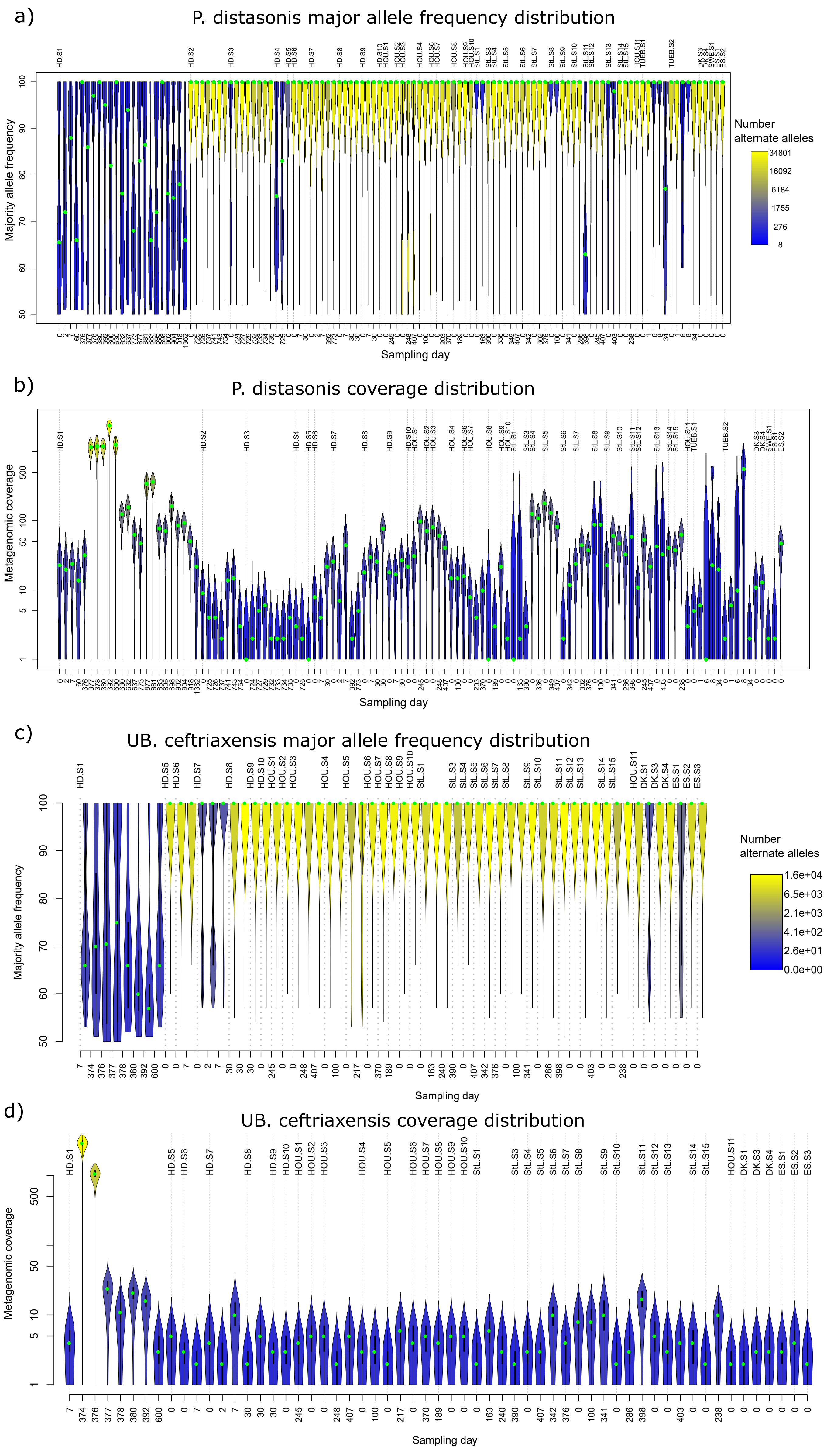

Suppl. Fig. 4: Coverages for each samples and major allele distribution for *P. distasonis*(a,b) and^{U}*B. ceftriaxensis*(c,d). a, c) The major allele frequency (MAF) can be used to infer the presence ofmultiple strains in metagenomic samples, if the MAF deviates significantly from 1. While for most samples MAF was on median 1 (green point), there were some samples with significantly deviations.These likely represent a single strain within a sample: as shown with the color code (representing closeness to the reference assembled from HD.S1) MAF depends strongly on the closeness to the reference genome, as large differences to the reference will shift the MAF distribution towards fixed (100% occurrence) alleles, obscuring the presence of very closely related strains. Thus, median MAF <<1 seems to represent in these samples within host diversity of a single strain population, as only few overallSNVs were present in these samples. Violin plots are colored by the number of alternate alleles discovered within a sample, as a proxy for difference to reference. b, d) Coverage distribution of single sites for the two reference genomes in each sample. This shows usually the expected coverage profiles, but note exceptions to this (e.g. b) Tueb.S1 samples). Violin plots are colored by average coverage of the respective genome. a-d) Above figure / data are the sample names of each subject involved in the study. Data was filtered for those samples with more than 3e6 total base coverage.

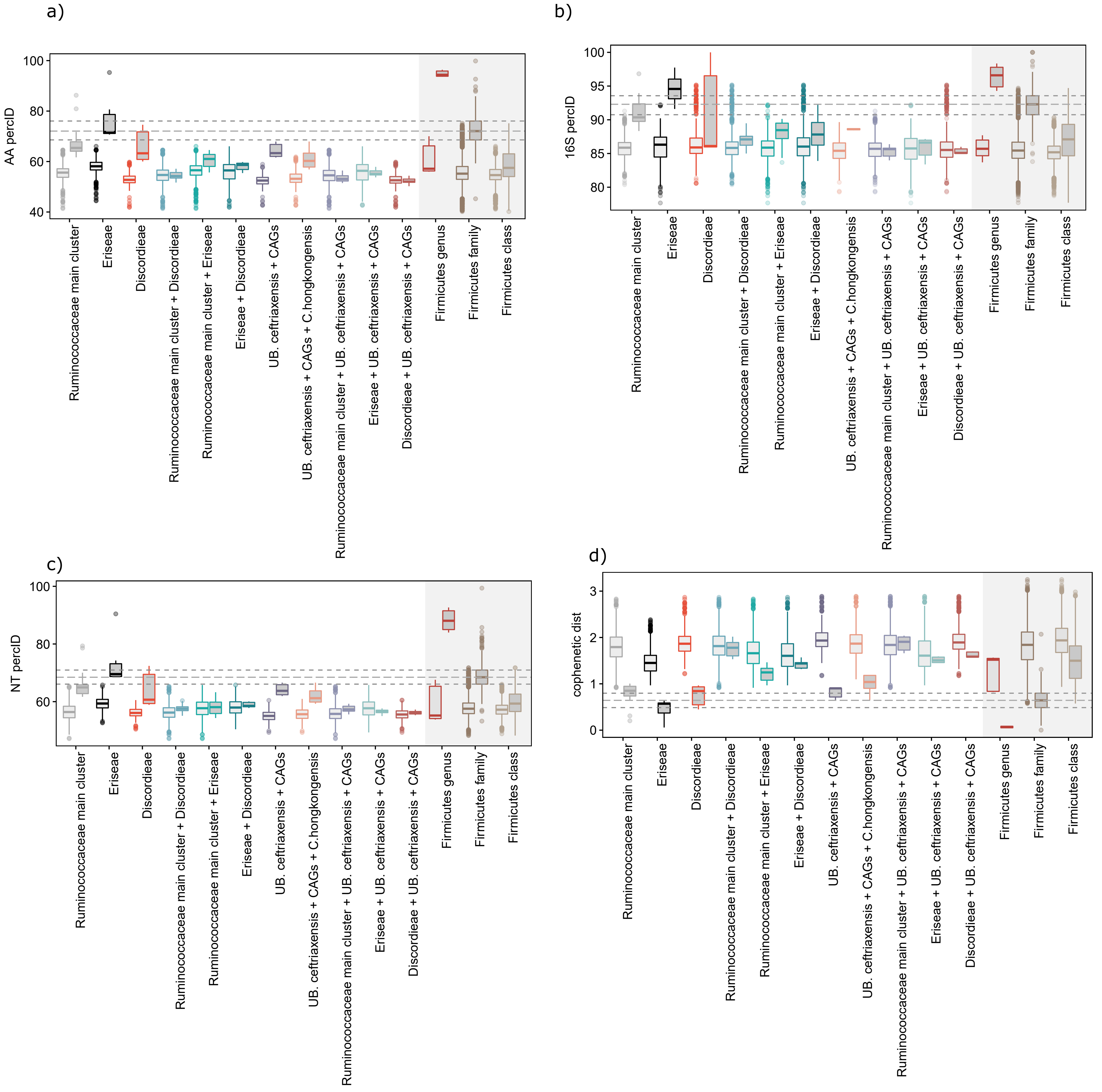

Suppl. Fig. 5: Genetic distance among firmicutes genomes within taxonomic groups (dark grey filled bars right) compared to other Firmicutes ("without", light grey filled bars left). This was inferred from a) the amino acid (AA) sequence of 40 conserved marker genes, b) the 16S nucleotide sequence, c) the nucleotide similarity between 40 conserved marker genes, and d) the cophenetic distance from the 40 marker genes tree (Fig. 3). As reference the median and upper/lower quantile of similarities of all Firmicutes families together is drawn via dashed lines, which shows the typical borders for an average firmicutes family. For all comparisons, a single genome representing a species was chosen. CAGs = Co-Abundance group, representing three genomes binned from metagenomics that are phylogenetically close to ^{U}*B. ceftriaxensis.*

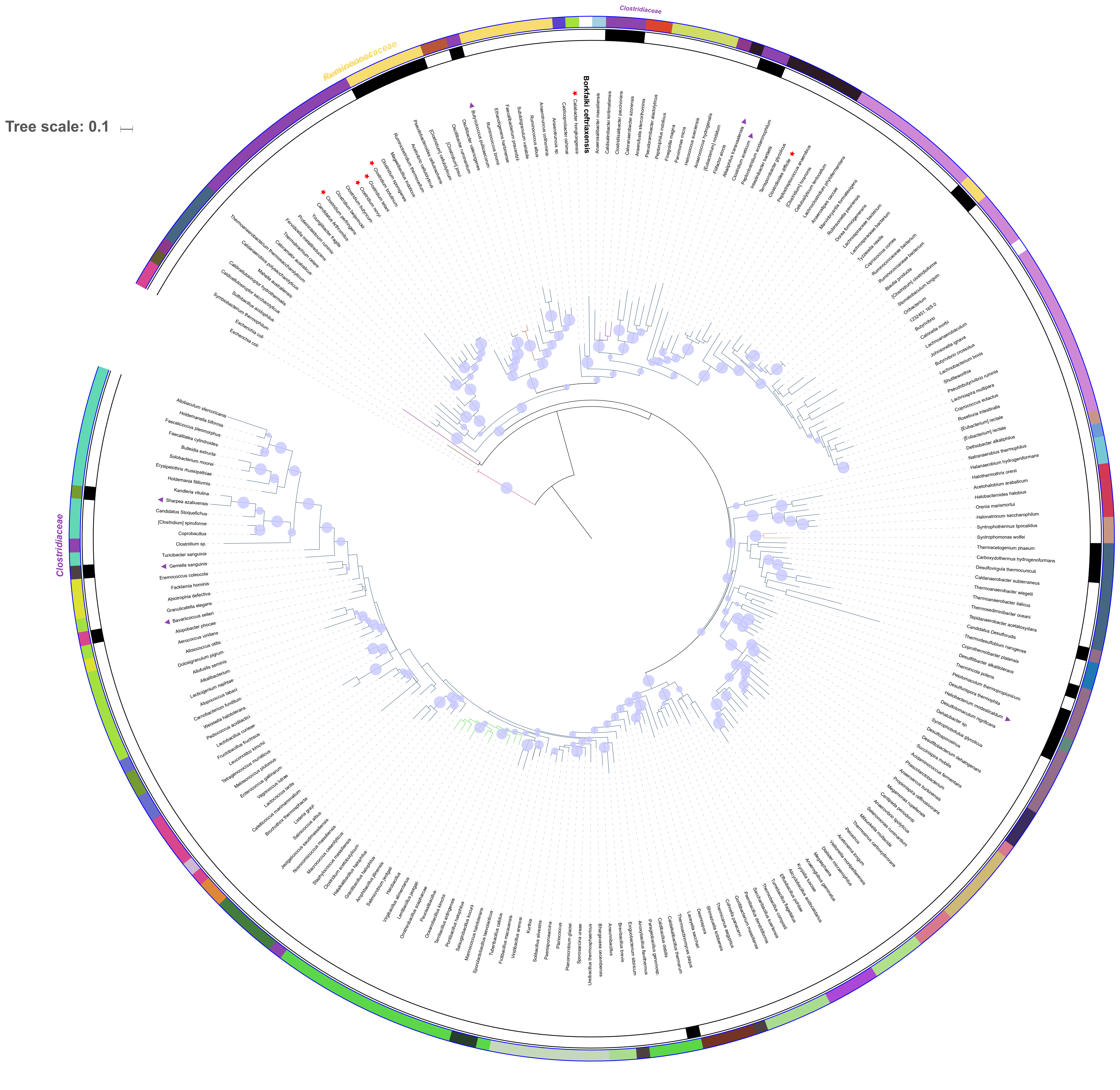

Suppl. Fig. 6: Phylogenetic maximum likelihood tree of species in Firmicutes reference database (see Methods), based on the nucleotidesequence of the 16S rRNA gene. ^{U}*B. ceftriaxensis*is in bold font. Note that for the three metagenomic binning (CAG's) that are closely related to ^{U}*B. ceftriaxensis*, no 16S rRNA gene is available.Bootstrap values >80 are demarked by blue circles and scaled in size. The outer ring color shows family assignments of the species in the tree. All families that were monophyletic are collapsed, while remaining families were either paraphyletic or the placement seemed not to fit a monophyletic family origin. Red stars mark pathogenic speciesor families containing pathogens, species that we propose to be placed in a different family are marked with purple triangles. IS = incertae sedis.

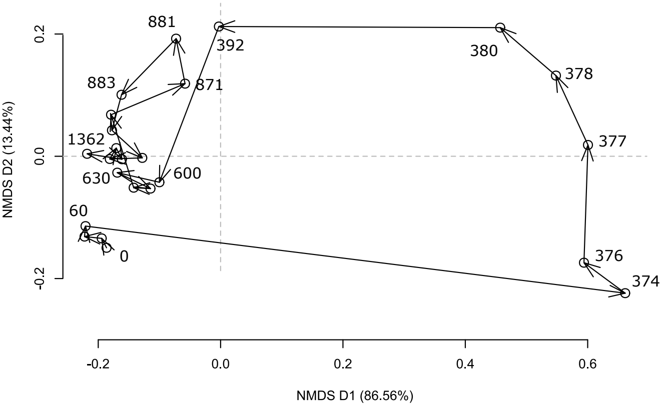

Suppl. Fig. 7: Taxonomic composition (species level) changesin subject HD.S1 over time as visualized with aNon-metric multidimensional scaling (NMDS) ordination. Numbers indicate the day in the time series.

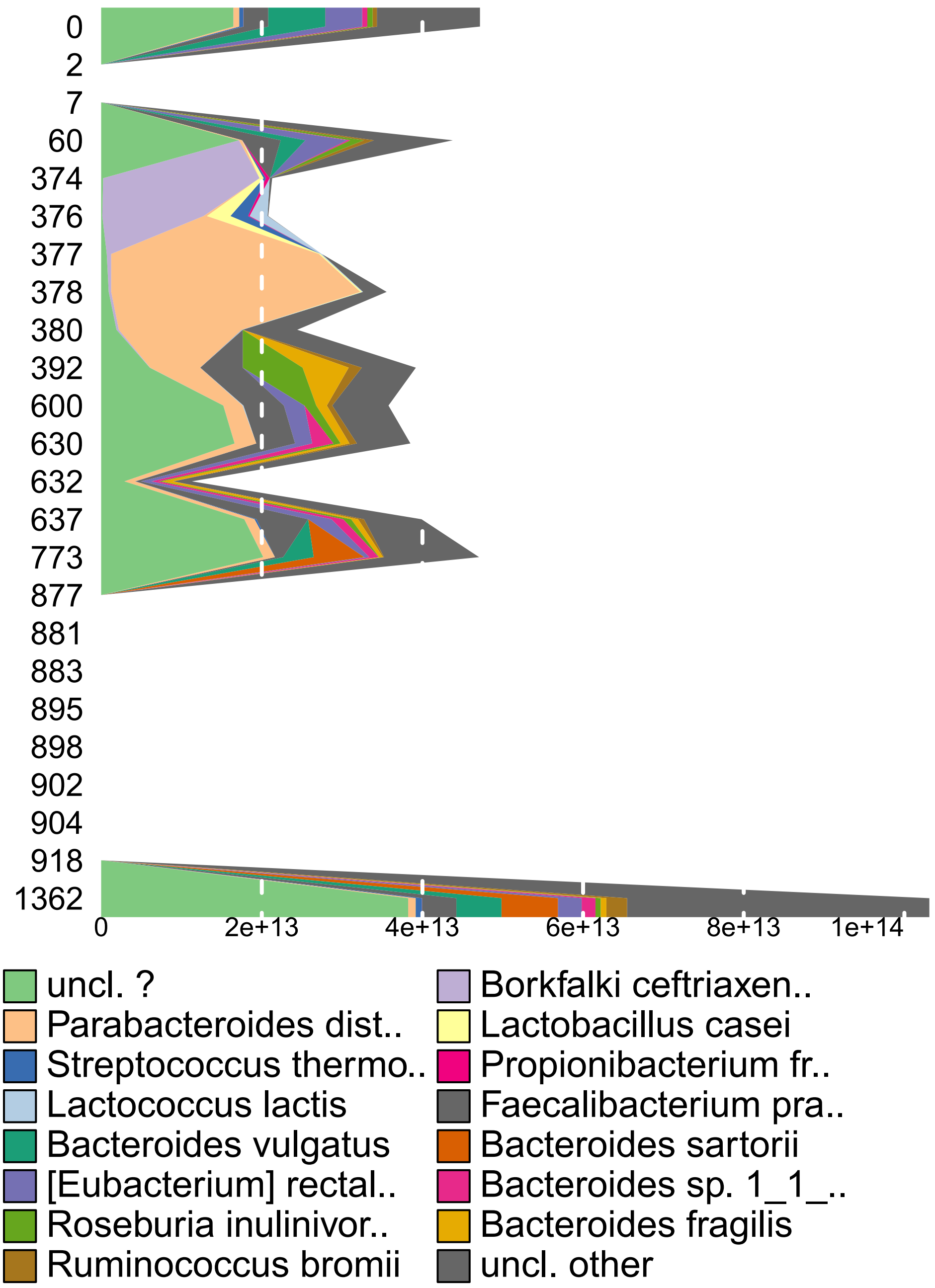

Suppl. Fig. 8: Composition of HD.S1 timeseries, normalized by actual cell counts. This shows the enrichment in absolute cell counts for ^{U}*B. ceftriaxensis* and *P. distasonis* during phases of monodominance. The high cell counts at day 1362 might be related to this sample being freshly analyzed, compared to older samplesthat were frozen before.

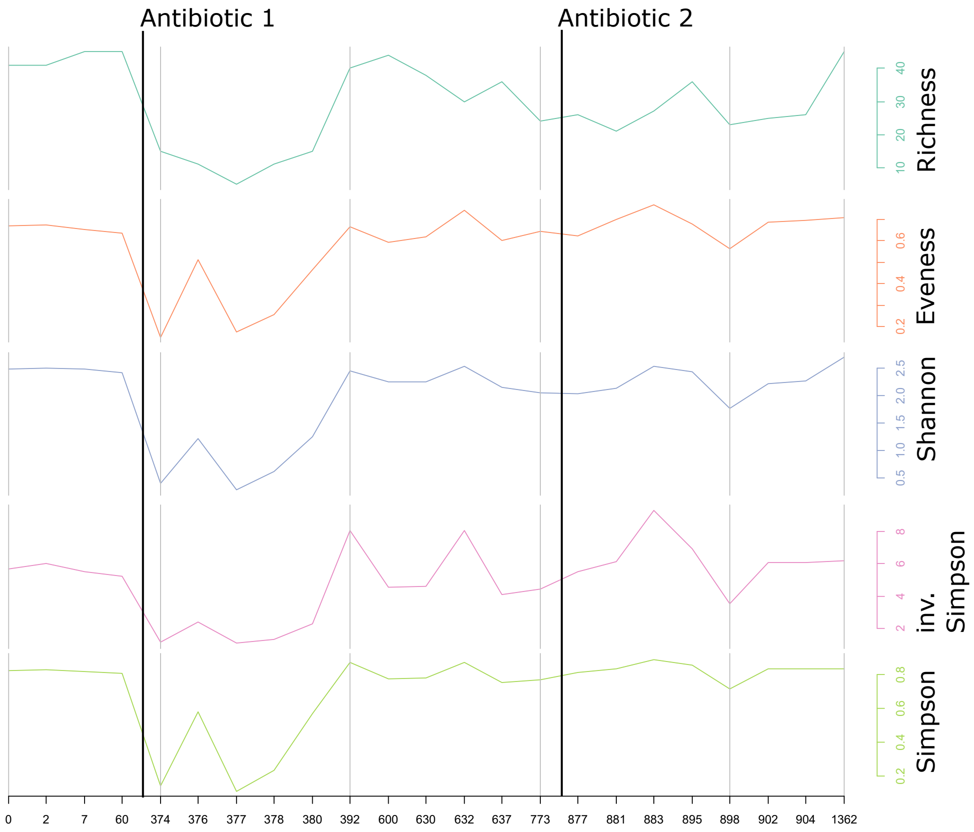

Suppl. Fig. 9: Different diversity measures in HD.S1. After antibiotic treatment on day 370 the community changes to a monodominant community state on days 374 (characterized by ^{U}*B. ceftriaxensis*) and day 377 (characterized by *P. distasonis*). Although the community diversity measurements are recovering in the following days and are less affected by the second antibiotic treatment on day 875, the community composition was changed until day 1362. Data was rarefied to 1,200-fold totalmarker gene coverage.

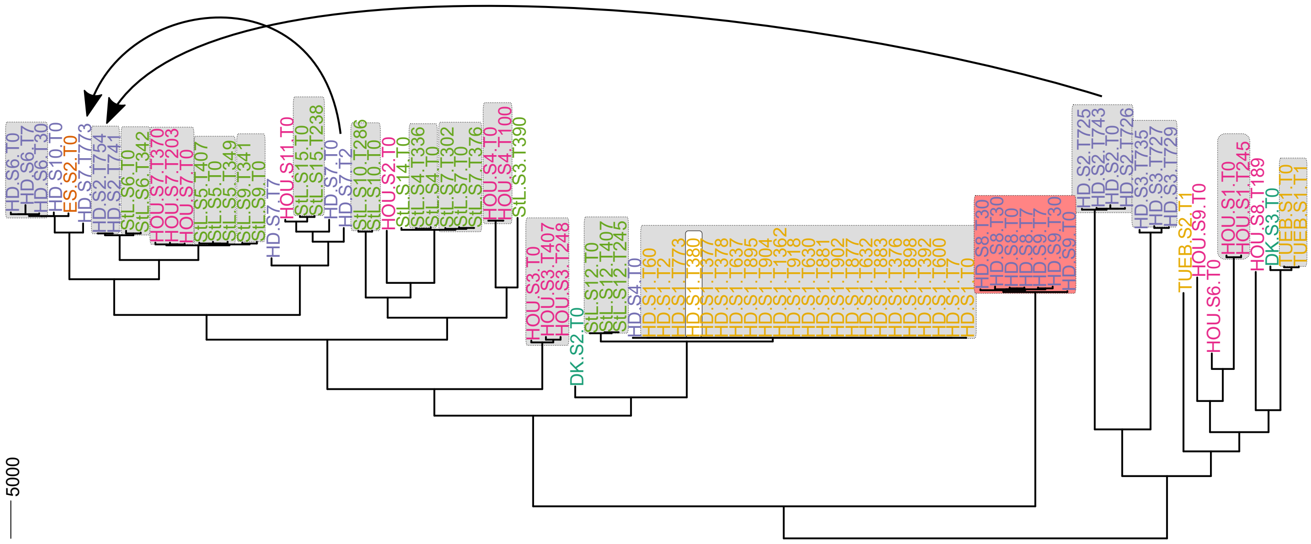

Suppl. Fig. 10: Reconstructed strains of *P. distasonis*comparedacross metagenomic samples. Nucleotide similarity was comparedacross the reconstructed genome sequence of *P. distasonis*for each metagenomic sample. Grey boxes demark samples from the same patient, showing that usually the specific genotype is stable within a patient. However, in two subject genotype exchanges are observed (black arrows). Note that HD.S9,HD.S8 and HD.S1,HD.S4 are individuals of the same family, respectively. The type strain (*P. distasonis*HDS1380) is based on the metagenomic assembly of HD.S1.T380 andis marked by a white box. The red box demarks *P. distasonis*strains of two individuals (HD.S8, HD.S9) that could not be clearly separated using this method.

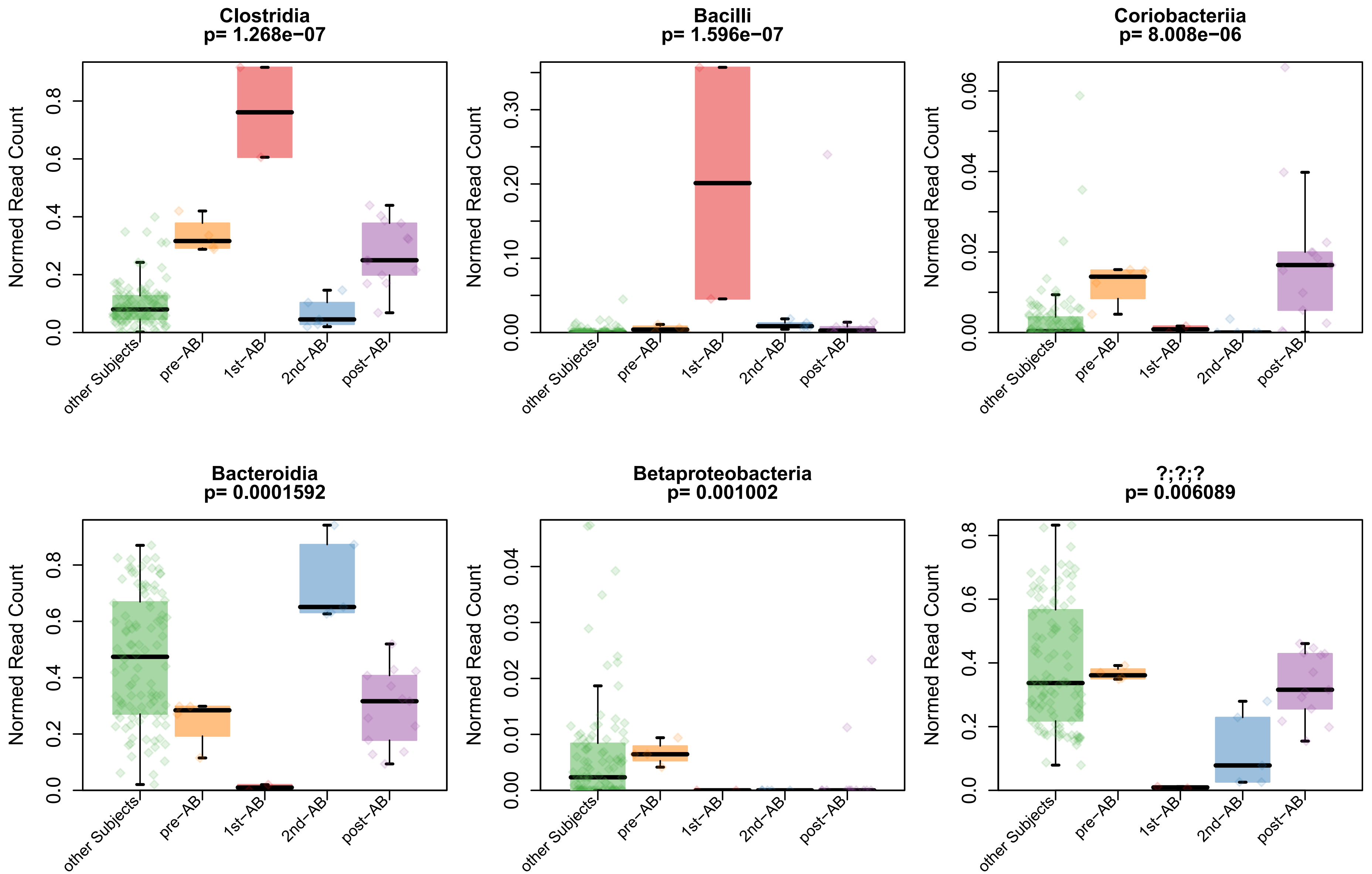

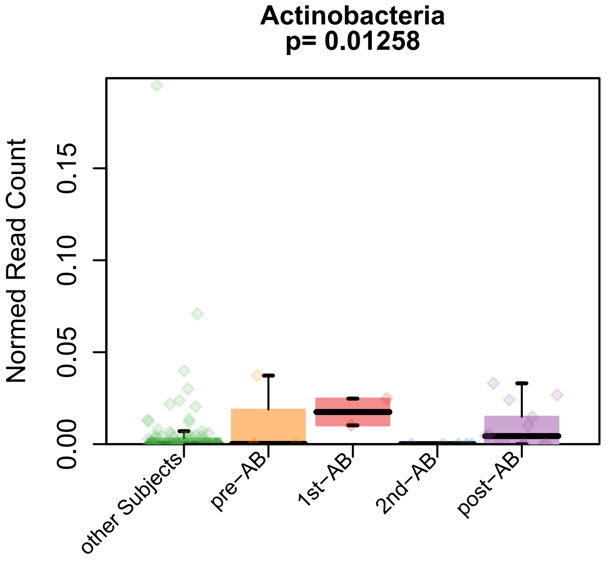

Suppl. Fig. 11: Class level differences between non-HD.S1 subjects in the study ("other subjects") and dynamics within HD.S1 upon antibiotic treatment, with 1st-AB referring to the first responsestagesamples after Ceftriaxone treatment (n=2) and 2nd-AB referring to the second response stage of the community (n=5), pre-AB (n=4) and post-AB (n=13) samples being all other HD.S1 samples. All classes are q<0.05 after multiple testing correction.

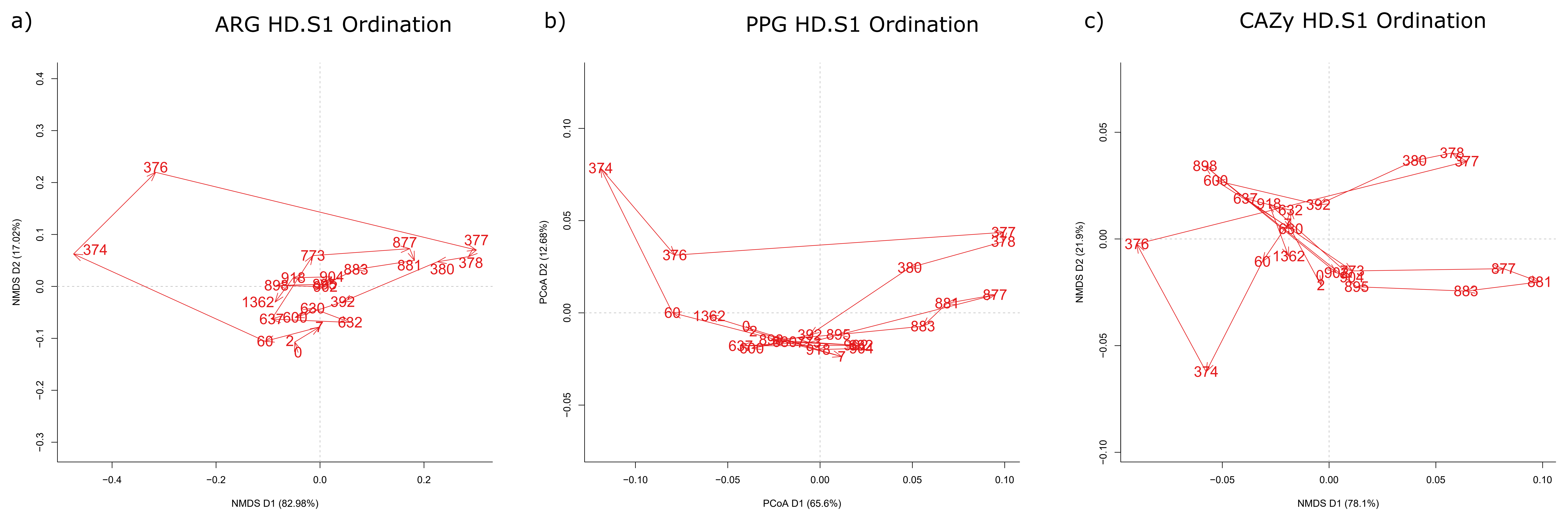

Suppl. Fig. 12: Ordination of a) antibiotic resistance genes (ARG), b) putative pathogenic/invasiongenes (PPG) and c) carbohydrate active enzyme (CAZy) compositional space in subject HD.S1 (Bray-Curtis intersample distance). Numbers represent the day of sampling. HD.S1 was treated on two occasions with antibiotics (before 374 and 877), and interruptions to the ARG composition are visible in each of these. Barplots on the sides are pearson correlations of different ARG classes to the axis 1 and 2 in the PCoA (a,b) and NMDS (c).

a)

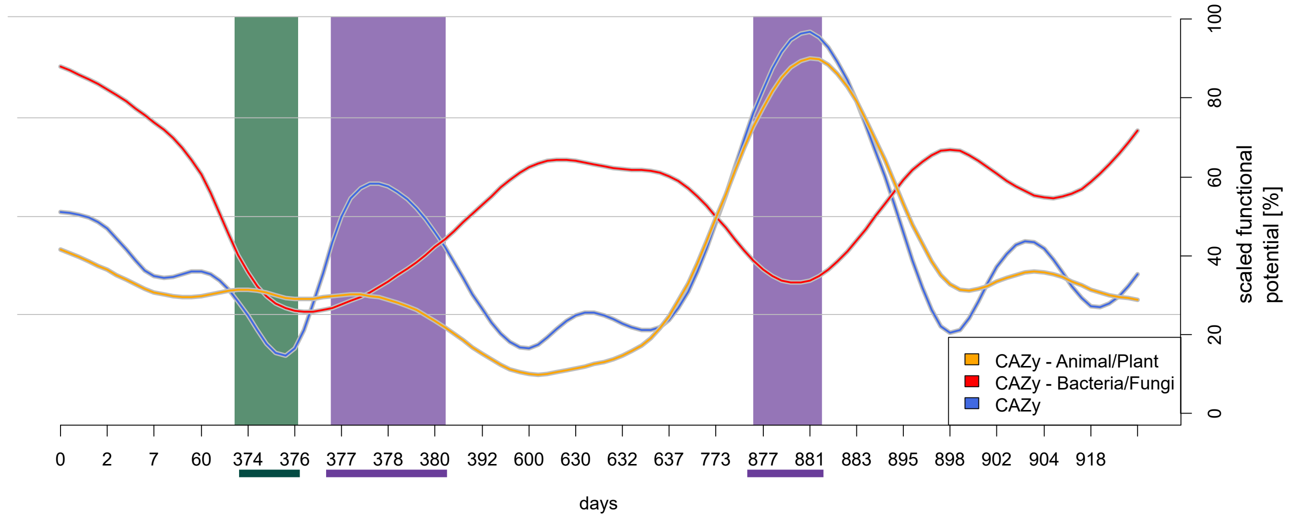

b)

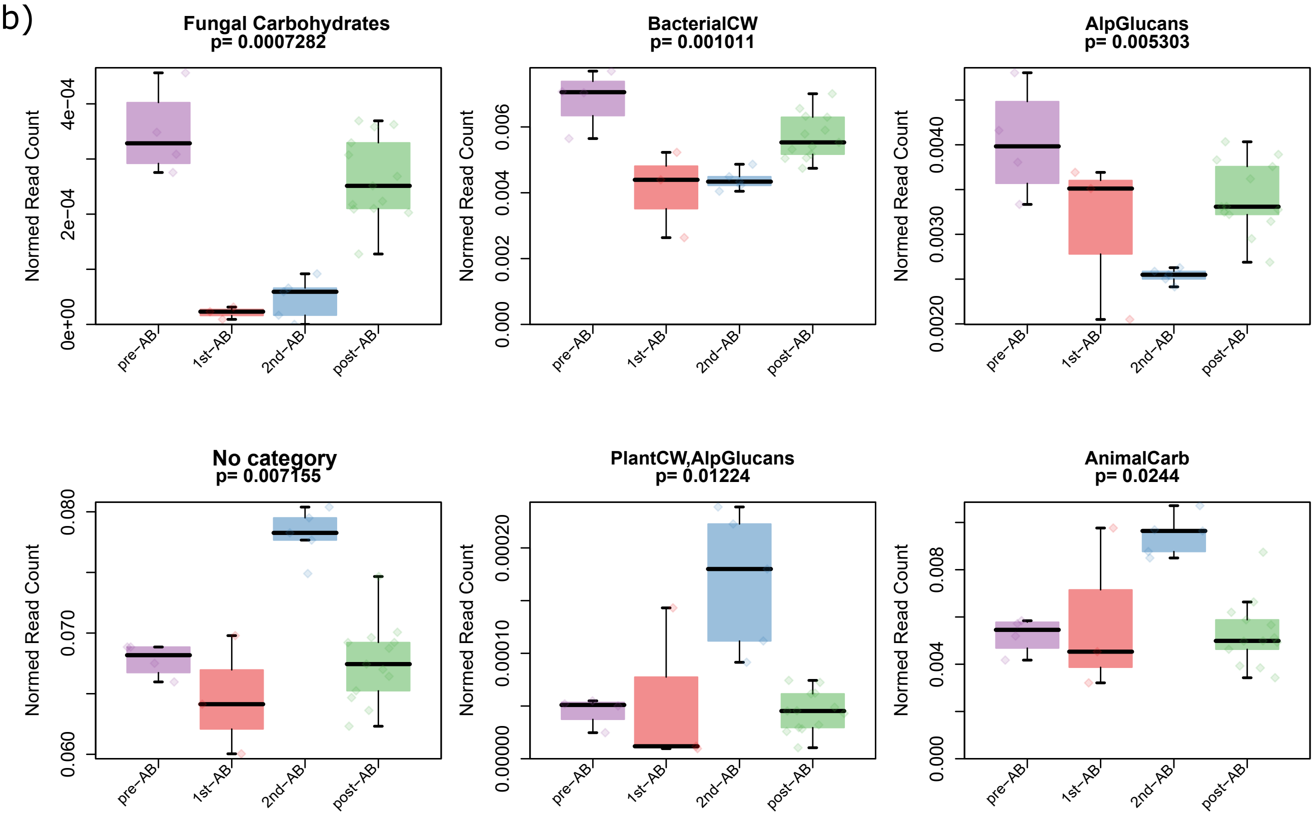

Suppl. Fig.13: Carbohydrate active enzyme (CAZy) abundance over time in HD.S1. a) The abundance of overall carbohydrate active enzymes (CAZy) genes (blue), the cumulative sum of subcategories specialized on bacterial + fungal derived carbohydrates (red), that are mostly based on resources available from members in the community, as well as carbohydrates derived from food sources (orange), animals and plants. The first (green) and second (purple) response stage are marked in the time series (as in Fig. 4). All categories are scaled by max/min abundance in HD.S1. b)There were significant differences for CAZyme genes in different substrate specificities during the time series in subject HD.S1 all tested categories are q<0.1 after multiple testing correction. Of note is that CAZymes specific to bacterial cell wall (CW) and fungal derived carbohydrates were significantly decreased during the both antibiotic response phases, while especially in the secondary antibiotics response CAZymes specific to food derived substrates were increased.Tested categories are defined in Suppl. Fig. 11.

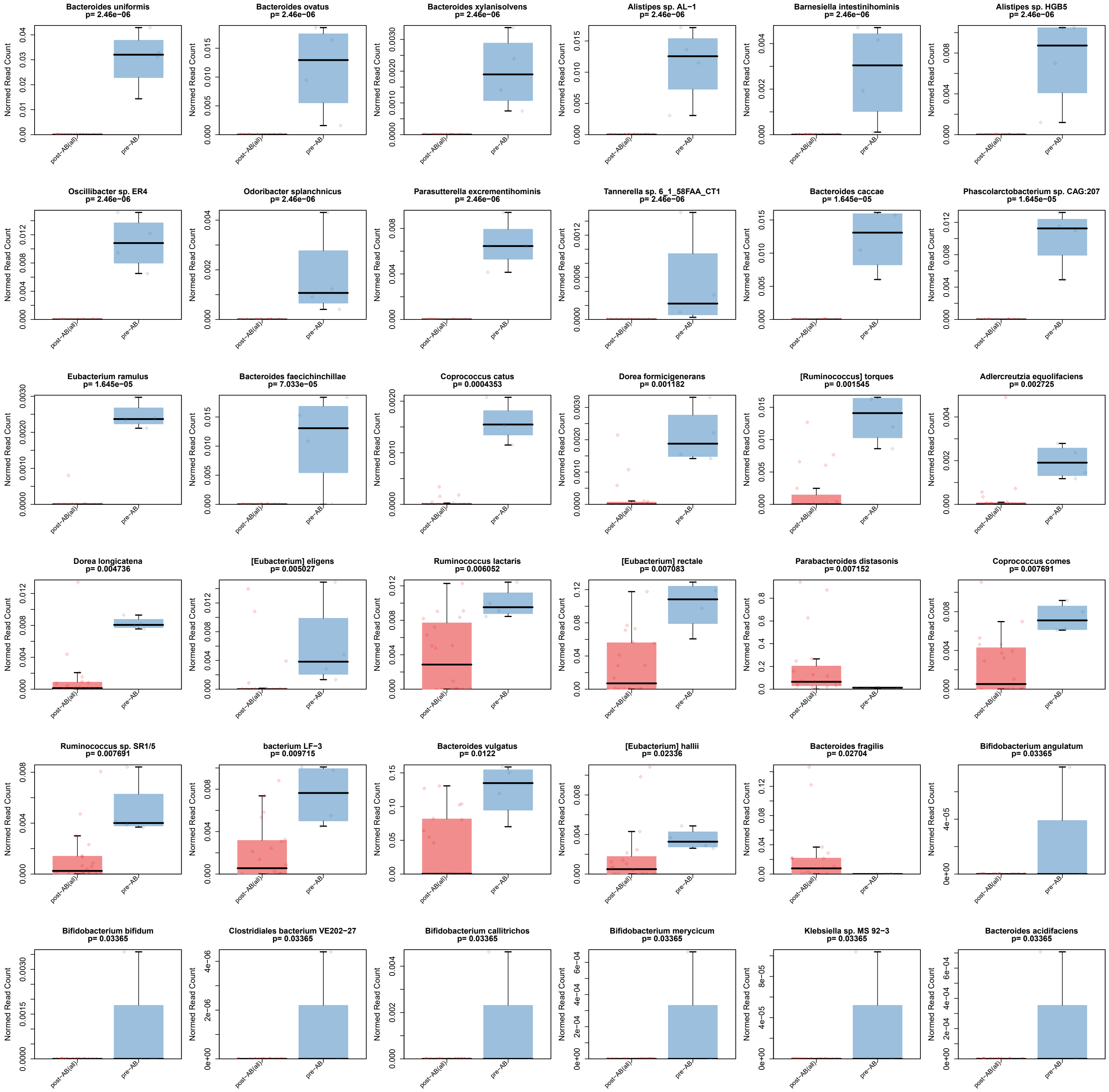

Suppl. Fig. 14: Species that are significantly different in abundance in subject HD.S1 between 4 pre-and 20 post-antibiotic treatment samples, the latter category also including samples during the treatment(Kruskal-Wallis testP-value).The first 25 species are significantly different after multiple testing correction (q<0.1).

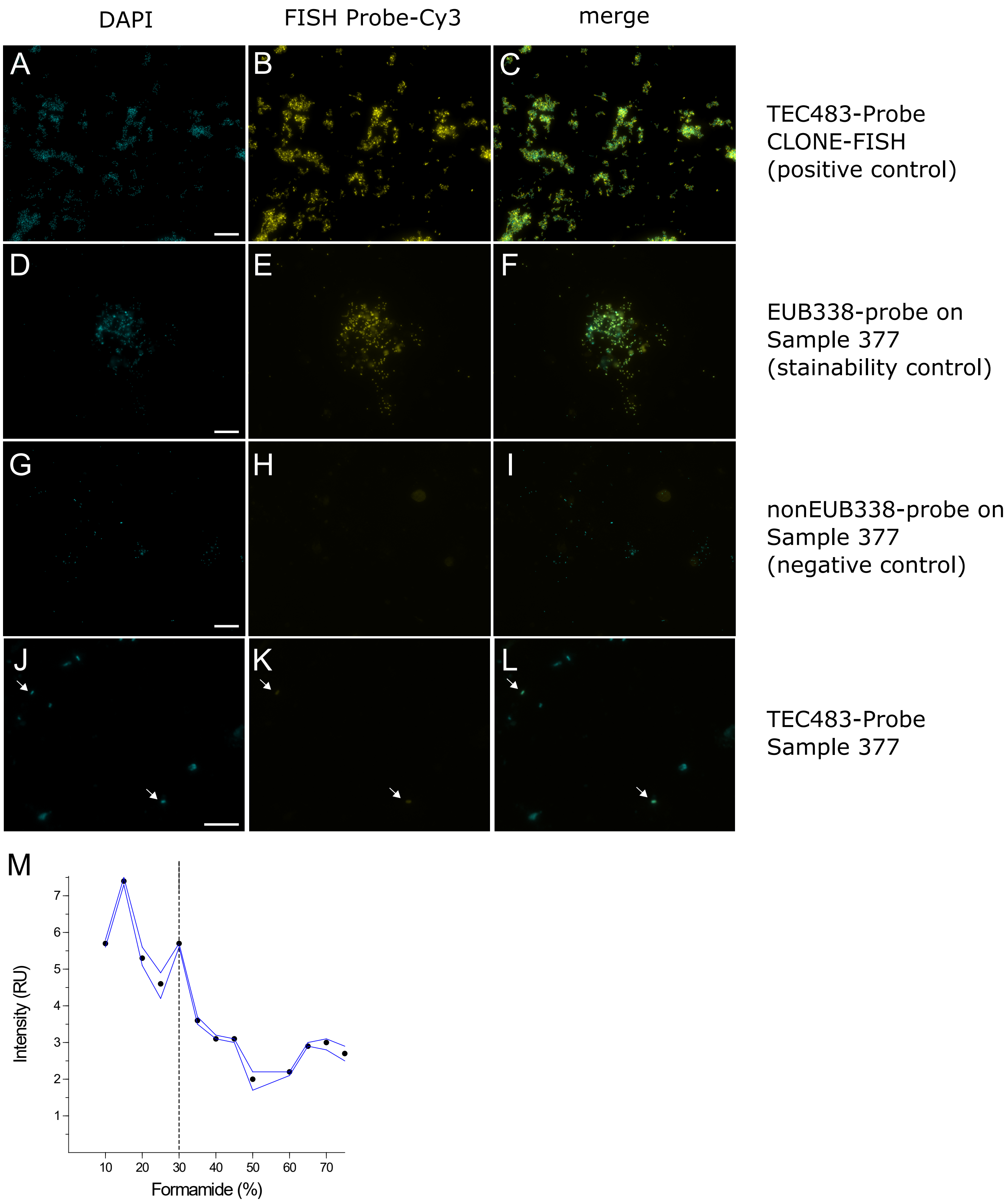

Suppl. Fig. 15.FISH-control experiments. (A-C) Sensitivity of TEC483 probe for the 16S rRNA-target was validated by CLONE-FISH where the target was heterologously transcribed in Escherichia coli.
(D-F) Stainability of previously frozen fecal samples upon hybridizationwith the domain-specific probe EUB338 staining most bacteria. (G-I) NonEUB338 control probe complementary to EUB338 was applied on the same sample (J-L) TEC483 probe signal for sample 337 arrows indicate positive cells (M) Formamide series using 10-75% FA to determine optimal hybridizationconditions. Scale Bar A-I 20µm, J-K 10µm

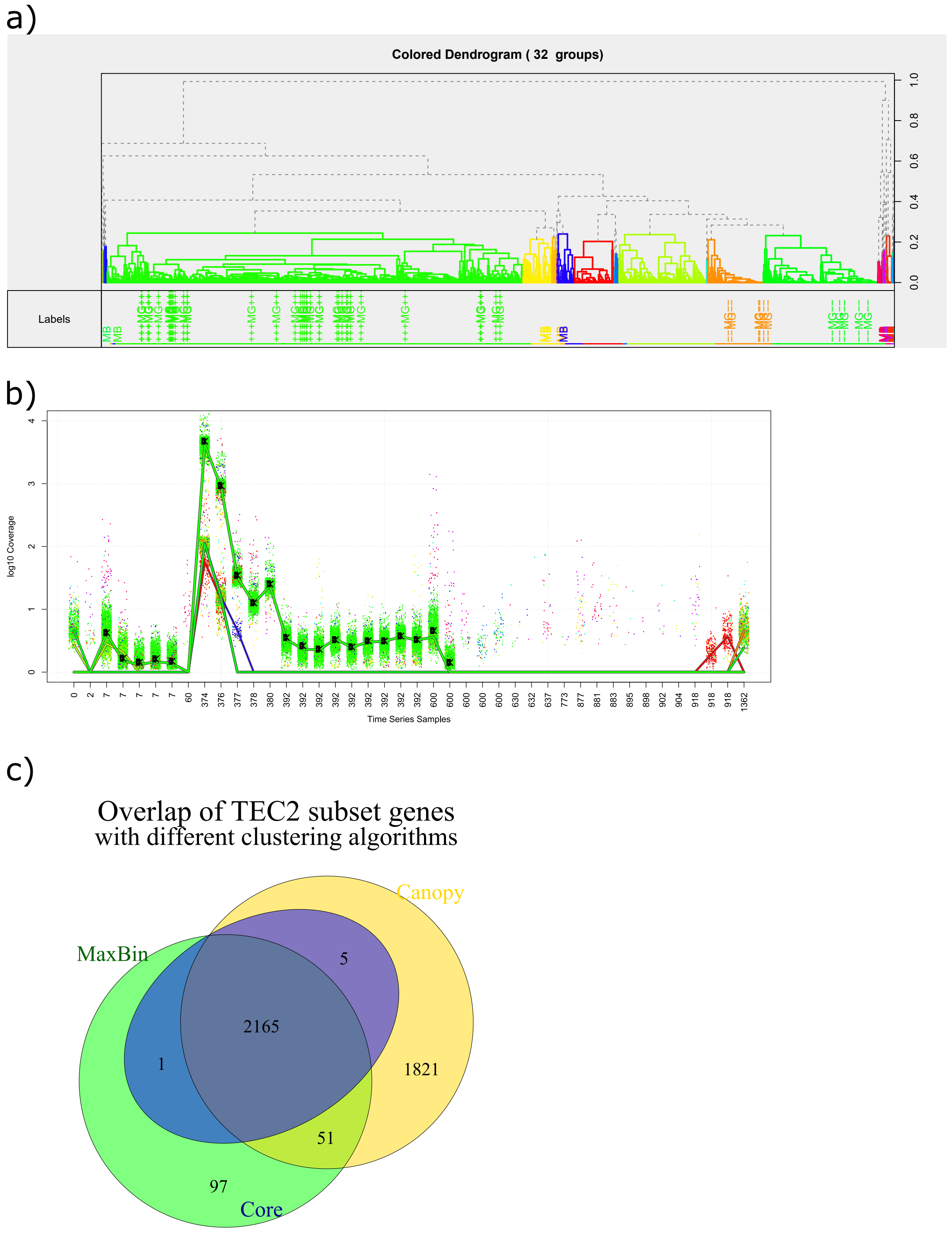

Suppl. Fig. 16: Genome Binning of ^{U}*B. ceftriaxensis*. a) The predicted genome bins from the Canopy clustering and MaxBin algorithms were included and tested for their correlation amongstthe subset of HD.S1 samples (shown in b). The correlation matrix was clustered with a hierarchical clustering and the cluster chosen that contained all 40 marker genes. Marker genes that occur in both genome bins are marked by “+++MG+++”, maker genes found in only one bin are marked by “—MG—“. Note that for the binning biological replicates, treated with different DNA extraction protocols, are included as separate samples. c) The final genes in the genome bin are compared to initialstarting set in a Venn diagram. This set of genes was further supplemented by co-assembled genes from mate pair sequencings, to get to the final genome with 2386 genes.

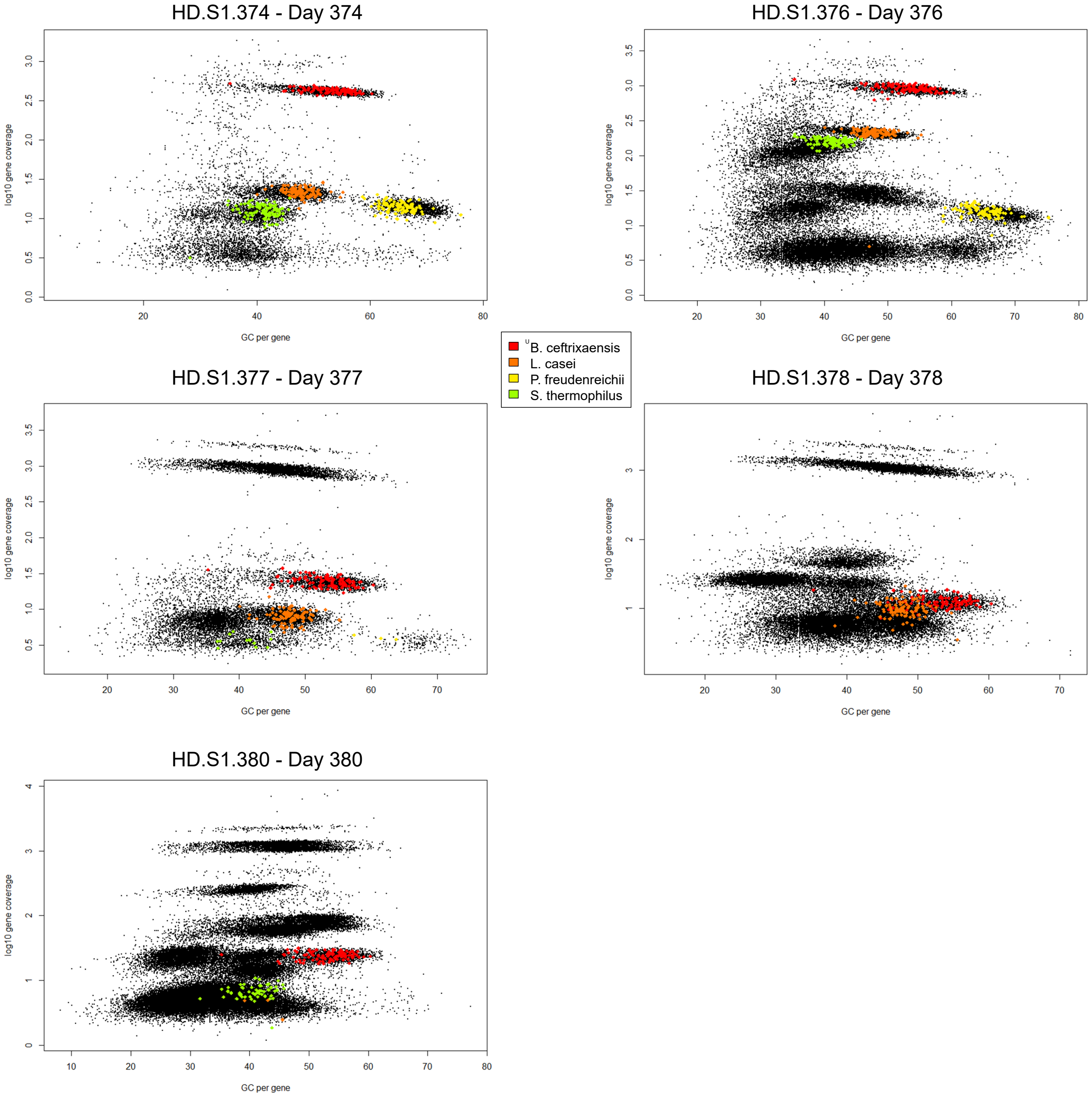

Suppl. Fig. 17:Tracking of species prevalent in HD.S1.374. In order to illustrate the changes in abundance after the ceftriaxone treatment, we show that abundance of gene clouds between day 374 –380 of patient HD.S1. The log10 transformed abundance and their respective GC content is shown for single genes (black points). Marker genes for ^{U}*B. ceftriaxensis*, *L. casei*, *P. freudenreichii*and *S. thermophilus*are shown as colored points.The unmarked high abundance gene cloud on day 377, 378 and 380 corresponds to the genome of *P. distasonis*.
