# Supplementary material for: Antibiotics-induced monodominance of a novel gut bacterial order
Source: Gut. 2019 Jan 18;68(10):1781–90. doi: 10.1136/gutjnl-2018-317715 (PMC6839795; doi:10.1136/gutjnl-2018-317715)
Supplement: Supplementary file 4 [file gutjnl-2018-317715supp004.pdf]

Supplemental Tables

**16S UB. ceftriaxensis Specific primer**									
	Sequence (5'->3')	Template strand	Length	Start	Stop	Tm	GC%	Self complementarity	Self 3' complementarity
Forward primer	GGCAAGCGTTATCCGGAATG	Plus	20	511	530	59.7	55	6	2
Reverse primer	GTAGTAGCGCCGAGGTTGAG	Minus	20	605	586	60.3	60	4	0
Internal oligo		Plus							
Product length					94				

Suppl. Table 2: 16S primers to detect ^{U}*B. ceftriaxensis*. 16S specific primers used for culturing experiments of ^{U}*B. ceftriaxensis*, constructed with NCBI primer blast.

	**^{U}*B. ceftriaxensis***	***P. distasonis***
Assembly size	2608799	4884146
Estimated genome completeness (%)	94.76	99.42
Estimated contamination (%)	2.45	0.38
N50	1925355	375178
Number of scaffolds	8	29
Average GC content (%)	51.67	44.95
Number of CDS	2386	4185
Number of rRNA genes	3	12
Number of tRNA genes	45	68

Suppl. Table 3: Assembly statistics. For the two bacterial genomes reconstructed from metagenomes in this study, assembly statistics a good quality.

**Object**	**Length (µm)**	**Width (µm)**
**cell1**	1.89	0.78
**cell2**	1.15	0.66

**cell3**	1.35	0.85
**cell4**	1.36	0.82
**cell5**	1.64	0.95
**average**	1.478	0.812

Suppl. Table 5: Cell size of new species. [U]*Borkfalki ceftriaxensis* size measurements based on FISH staining of fecal samples.

group	median AA percID	median NT percID	median percID 16S	minimum AA percID	minimum NT percID	minimum percID 16S
**all Ruminococcaceae**	60.71	59.33	88.81	51.67	54.32	83.67
**Ruminococcaceae main cluster**	65.40	64.94	90.36	61.66	61.68	88.33
**Ruminococcaceae main cluster + Eriseae**	61.03	58.09	88.46	55.67	54.32	83.67
**Ruminococcaceae main cluster + Discordieae**	54.23	57.54	87.09	51.67	55.10	85.00
**Eriseae**	71.73	69.56	94.57	70.71	68.42	91.61
**Discordieae**	63.27	60.68	86.11	60.02	58.89	85.93
**Eriseae + Discordieae**	58.66	58.55	87.82	55.43	57.80	83.89
**UB. ceftriaxensis + CAGs**	63.33	63.75	NA	61.60	61.94	NA
**UB. ceftriaxensis + CAGs + C.hongkongensis**	60.25	61.20	88.60	56.86	59.26	88.44
**UB. ceftriaxensis + C.hongkongensis**	56.86	59.86	88.60	56.86	59.86	88.44
**Ruminococcaceae main cluster + UB. ceftriaxensis + CAGs**	53.09	57.31	85.65	51.46	55.58	83.98
**Eriseae + UB. ceftriaxensis + CAGs**	55.05	56.76	86.62	53.73	55.01	83.38
**Discordieae + UB. ceftriaxensis + CAGs**	52.04	56.14	85.08	50.22	55.30	85.00
**Firmicutes within genus**	94.79	88.23	96.39	93.61	83.98	94.31
**Firmicutes within family**	70.90	67.92	91.56	45.25 (60.9)	53.25 (60.51)	84.44 (88.68)
**Firmicutes within class**	62.06	61.58	88.23	55.02	57.29	85.23

Suppl. Table 9: Genetic similarity of taxonomic groups within Firmicutes. Genetic distance among Firmicutes genomes within taxonomic groups indicated as median and minimum sequence identity of the the amino acid (AA) sequence of 40 conserved marker genes, the nucleotide similarity (NT) between 40 conserved marker genes and the 16S nucleotide sequence. In brackets the minimum sequence identity without the family Thermodesulfobiaceae is shown as it strongly deviates from all other families.

Species	Current family	Suggested family	Supported 40AA	Supported 40nt	Supported 16S	
						**Reassign family**
**Hungatella hathewayi**	Clostridiaceae	Lachnospiraceae	Y	Y	Y	
**Heliobacterium modesticaldum**	Heliobacteriaceae	Peptococcaceae	Y	?	Y	
**Bavariicoccus seileri**	Enterococcaceae	Carnobacteriaceae	Y	Y	Y	
**Sharpea azabuensis**	Lactobacillaceae	Erysipelotrichaceae	Y	Y	Y	
**Alkaliphilus transvaalensis**	Clostridiaceae	Peptostreptococcaceae	Y	Y	Y	
**Clostridium aceticum**	Clostridiaceae	Peptostreptococcaceae	Y	Y	Y	
**Butyricicoccus pullicaecorum**	Clostridiceae	Ruminococcaceae/ or Oscillospiraceae	Y	Y	Y	
						**Create new family**
**Coprothermobacter platensis**	Thermodesulfobiaceae	?	Y	Y	Y	
**Clostridium cellulyticum**	Ruminococcaceae	Erisaceae	Y	Y	Y	
**Clostridium josui**	Ruminococcaceae	Erisaceae	Y	Y	Y	
**Pseudobacteroides cellulosolvens**	Ruminococcaceae	Erisaceae	Y	Y	Y	
**Ruminoclostridium thermocellum**	Ruminococcaceae	Erisaceae	Y	Y	Y	
**Acetivibrio cellulyticus**	Ruminococcaceae	Erisaceae	Y	Y	Y	
***Ruminococcaceae bacterium* AB4001**	Ruminococcaceae	Discordiaceae	Y	Y	Y	
***Ruminococcaceae bacterium* AE2021**	Ruminococcaceae	Discordiaceae	Y	Y	Y	
Mageeibacillus indolicus	Ruminococcaceae	Discordiaceae	Y	Y	Y	
**Caldisalinibacter kiritimatiensis**	Clostridiaceae	?	Y	Y	Y	
**Clostridiisalibacter paucivorans**	Clostridiaceae	?	Y	Y	Y	

**Caloranaerobacter azorensis**	Clostridiaceae	?	Y	Y	Y	

Suppl. Table 10: Restructured Firmicutes phylogeny. Proposed changes to current Firmicutes taxonomy, based on 40 marker genes (AA or nt sequence) or 16S rRNA gene based maximum likelihood phylogenies.

**ARG**	**p-value**	**Enrichment**	**PostHoc test**	**2nd-AB - 1st-AB**	**post-AB - 1st-AB**	**pre-AB - 1st-AB**	**post-AB - 2nd-AB**	**pre-AB - 2nd-AB**	**pre-AB - post-AB**
	0.001077	pre-AB = 2nd-AB>> post-AB > 1st-AB	q-val	0.066806723	0.042857143	0.068571429	0.00140056	0.904761905	0.066806723
			p-val	0.035714286	0.014285714	0.057142857	0.000233427	0.904761905	0.044537815
**PPG**	p-value	Enrichment	PostHoc test	2nd-AB - 1st-AB	post-AB - 1st-AB	pre-AB - 1st-AB	post-AB - 2nd-AB	pre-AB - 2nd-AB	pre-AB - post-AB
	0.037756	1st-AB = pre-AB = post-AB > 2nd-AB	q-val	0.685714286	0.685714286	0.857142857	0.026610644	0.095238095	0.260504202
			p-val	0.571428571	0.521428571	0.857142857	0.004435107	0.031746032	0.130252101

Suppl. Table 12: Significant changes in overall PPG and ARG potential between antibiotic treatments. Kruskal-Wallis test and posthoc test on the total ARG (antibiotic resistance gene) and PPG (putative pathogenicity gene) abundance between different phases in HD.S1 time series. 1st-AB refers to the first response stage samples after Ceftriaxone treatment (n=2) and 2nd-AB referring to the second response stage of the community (n=5), pre-AB (n=4) and post-AB (n=13) samples being all other HD.S1 samples. Enrichment columns shows the median class abundance for each feature sorted descending, with “=” indicating no significant difference between adjacent labels and "<", "<<", "<<<" indicating a significant difference at q<0.1, 0.05, 0.01, respectively, in a post-hoc test.
